# Deep learning-based biliary stent classification and transfer learning adaptation to an additional stent type

**DOI:** 10.1186/s41747-026-00749-4

**Published:** 2026-06-08

**Authors:** Jin Lee, Byeong-Kwon Shin, Gwang-Hyun Yu, Thanh Vu Dang, Chang Jin Yoon, Jae Hwan Lee, Chong-Ho Lee, Young-Min Han, Jin-Young Kim, Kun Yung Kim

**Affiliations:** 1 Department of Intelligent Electronics and Computer Engineering, Chonnam National University, Gwangju, Republic of Korea; 2 Department of Radiology, Jeonbuk National University Hospital, Jeonju-si, Republic of Korea; 3 Research Institute of Clinical Medicine of Jeonbuk National University, Biomedical Research Institute of Jeonbuk National University Hospital, Jeonju-si, Republic of Korea; 4 Research Center, AISeed Inc., Gwangju, Republic of Korea; 5 Department of Radiology, Seoul National University College of Medicine, Seoul National University Bundang Hospital, Seongnam-si, Republic of Korea

**Keywords:** Artificial intelligence, Biliary tract, Deep learning, Diagnostic imaging, Self expandable metallic stents

## Abstract

**Objective:**

Accurate classification of previously deployed biliary stents is crucial for planning reintervention, yet conventional image interpretation is challenging due to diverse vendor-specific designs. This study aimed to develop a deep learning model for biliary stent classification and to evaluate transfer learning as an adaptation strategy for a new stent type.

**Materials and methods:**

This single-center study included 185 patients who underwent biliary stent placement. The primary dataset (412 images from 151 patients) included four stent types: Epic™, EGIS, Niti-S, and Bonastent® uncovered. The augmented dataset (488 images from 185 patients) incorporated additional images of Bonastent® partially covered stents. A ResNet-50 model was trained to classify stents by number (single *versus* multiple), vendor, and specific type using 5-fold cross-validation. Transfer learning was applied to incorporate the additional stent type into the model. Accuracy, precision, recall, and F1 score were calculated as mean ± standard deviation across folds.

**Results:**

For the primary dataset, the model achieved an F1 score of 57.03 ± 6.77 for single/multiple detection and 94.78 ± 4.07 for vendor classification. Stent-specific F1 scores ranged from 91.43 ± 3.43 (Bonastent® uncovered) to 97.91 ± 2.59 (Epic™). After augmentation, the model yielded similar or improved scores. Transfer learning achieved comparable results (*e.g*., 59.06 ± 9.08 for single/multiple, 83.6 ± 5.8 to 97.73 ± 2.42 for stent-specific).

**Conclusion:**

The ResNet-50 model demonstrated reliable performance in classifying biliary stents on radiographic and fluoroscopic images. Transfer learning allowed the model to be updated to incorporate newly introduced stent types with minimal degradation in performance.

**Relevance statement:**

Deep learning–based biliary stent classification may assist clinicians by rapidly classifying previously placed stents on radiographic and fluoroscopic images, thereby supporting procedural planning and device confirmation during biliary interventions.

**Key Points:**

- Accurate classification of previously deployed biliary stents remains difficult due to diverse vendor-specific designs and similar radiographic appearances.
- A ResNet-50 deep learning model reliably classified biliary stents, and transfer learning enabled the model to incorporate newly introduced stent types while maintaining performance comparable to the baseline model.
- This artificial intelligence-based approach provides rapid classification of previously placed biliary stents on routine images, which may assist with device verification during biliary interventions.

**Graphical Abstract:**

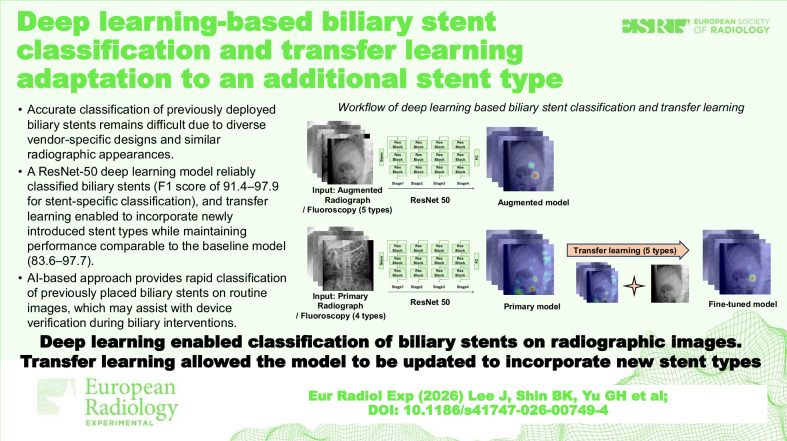

## Background

Self-expandable metallic stents (SEMS) have become the standard therapeutic modality for malignant biliary strictures caused by conditions such as cholangiocarcinoma, pancreatic cancer, and metastatic diseases [1–3]. The development of SEMS has led to the introduction of various stent designs, including uncovered, fully covered, and partially covered SEMS, each with its own unique advantages and drawbacks in clinical practice [4]. Uncovered SEMS, the most widely used type, effectively anchor to the tissue to minimize migration risk but remain susceptible to late occlusion. Conversely, fully covered SEMS prevent tissue ingrowth, yet pose greater risks of migration and may block side branches due to their covering membrane [2, 5, 6]. Partially covered SEMS aim to combine these advantages by covering the central portion of the stent while leaving both ends uncovered to anchor within the biliary duct [7–9].

Complex biliary anatomy, particularly in hilar strictures requiring the placement of multiple stents, further complicates stent selection [10]. Specific stent designs, such as uncovered SEMS with large-cell configurations, facilitate subsequent stent insertion through pre-existing meshes, whereas double-layered stents with denser mesh structures aim to minimize tumor ingrowth without sacrificing mechanical stability [11, 12]. Reintervention for biliary stent dysfunction remains common in clinical practice, and accurate recognition of previously deployed stent types can support appropriate procedural planning. However, recognizing previously placed stent types has become challenging due to numerous vendor-specific stent designs. Accurate classification of previously deployed stents can facilitate the selection of appropriate techniques and stent types during reintervention.

To overcome the limitations of conventional image interpretation, recent advances in artificial intelligence, particularly deep learning (DL) using convolutional neural networks (CNNs), have shown promising results in various medical domains [13]. CNNs autonomously extract complex, discriminative features directly from raw pixel data, enabling classification without requiring extensive manual feature engineering [14, 15]. Prior studies have demonstrated the feasibility of automated stent-graft segmentation during endovascular aortic repair using digital subtraction angiography or fluoroscopy, as shown by Kappe et al [16] and Breininger et al [17]. DL models have also achieved high performance in classifying dental implants [18]. These findings suggest the potential applicability of DL for stent classification in biliary interventions. In contrast to aortic stent-grafts or dental implants, biliary SEMS are substantially smaller in diameter, resulting in low signal-to-noise ratios and lower pixel occupancy. Furthermore, they exhibit structural complexity due to denser, heterogeneous mesh configurations and are often deployed in overlapping patterns. These characteristics make visual classification considerably more challenging, particularly during reintervention. Moreover, biliary interventions are frequently repeated, and manual review of prior medical records to confirm the originally deployed stent type is time-consuming and not always feasible in daily clinical settings. Given these domain-specific challenges, it is essential to verify whether DL algorithms can effectively extract discriminative features from such challenging images. Successfully establishing an automated classification system in this complex domain may provide meaningful advantages by offering rapid and objective identification of previously placed biliary stents.

However, a practical challenge arises when new stent variants are introduced into clinical practice, necessitating continuous updates to classification models. Complete retraining of CNN models for every newly introduced stent would demand substantial computational resources and could disrupt existing clinical processes. To overcome this limitation, transfer learning methods provide valuable solutions. These strategies allow efficient adaptation of pretrained models to new stent variants even when annotated data is limited, reducing training time and resources associated with model redevelopment [19, 20]. Therefore, this study aimed to evaluate a DL algorithm for biliary stent classification and to compare the performance of transfer learning with different training epochs in integrating new stent designs.

## Methods

### Dataset

This single-center retrospective study was approved by the Institutional Review Board of Jeonbuk National University Hospital, with a waiver for informed consent (number: 2025-03-007). Consecutive patients who underwent biliary stent placement from January 2018 to April 2023 were included in Table 1. All stents were placed *via* interventional procedures, except for the Bonastent® partially covered biliary stents, which were placed endoscopically. All plain abdominal radiographs and fluoroscopic images were anonymized, then converted into portable network graphics (PNG) format. One interventional radiologist with 10 years of experience identified and classified all biliary stents by referencing both the images and the verified procedure/device codes recorded at the time of deployment.

**Table 1:** Demographics of the dataset

	Primary set (*n* = 151)	Augmented set (*n* = 185)
Mean age (years)	74.3 ± 12.7	75.0 ± 12.2
Male	91 (60.3)	109 (58.9)
Cause of biliary stricture		
Bile duct cancer	97 (64.2)	115 (62.2)
Pancreas cancer	31 (20.5)	44 (23.7)
Other malignancy	21 (13.9)	23 (12.4)
Cholelithiasis	2 (1.3)	3 (1.6)
Biliary stent placement		
Percutaneous approach	151 (100)	151 (81.6)
Endoscopic approach	0 (0)	32 (17.3)
Biliary stent types		
Epic only	38 (25.2)	38 (20.5)
EGIS only	25 (16.6)	25 (13.5)
NITI-S only	59 (39.1)	59 (31.8)
Bona uncovered only	22 (14.6)	22 (11.9)
Bona partially covered only	0 (0)	32 (17.3)
Multiple stent types	7 (4.6)	9 (4.9)
Mean number of biliary stents per patient	1.1 ± 0.4	1.1 ± 0.4
Biliary stent-in-stent placement	12 (7.9)	15 (8.1)
Stents in other abdominal organs	8 (5.3)	12 (6.5)

Data are presented as means ± standard deviation or number of patients with percentages in parentheses

To evaluate both the classification performance and the effect of transfer learning, two datasets were constructed: a primary dataset and an augmented dataset. The primary dataset was used to develop and train the baseline deep learning model, providing the initial supervised label space for biliary stent classification. The augmented dataset incorporated additional images of newly introduced stent types to simulate a clinical scenario in which the classifier must be updated as new devices appear. This separation allowed assessment of baseline model performance as well as the feasibility of updating the model through transfer learning.

As a primary dataset, a total of 412 abdominal radiographs or fluoroscopic images from 151 patients were included. The mean patient age was 74.3 ± 12.7 years (mean ± standard deviation), and 60.2% of the patients were male. Among the 412 images, 144 were radiographs and 268 were fluoroscopic images. The dataset comprised the following biliary stents: Epic™ biliary stent (Epic, Boston Scientific: *n* = 113, 27.4%), EGIS biliary stent (EGIS, S&G Biotech, Inc.; *n* = 121, 29.4%), Niti-S biliary stent (Niti-S, Taewoong Medical Co., Ltd.; *n* = 170, 41.3%), and Bonastent® uncovered biliary stent (Bona UMS, Sewoon Medical Co., Ltd. South Korea; *n* = 64, 15.5%). The primary dataset was randomly divided into 5 sets to institute 5-fold cross-validation, with a training-to-test ratio of approximately 4:1.

As an augmented dataset, a total of 488 abdominal radiographs or fluoroscopic images, including an additional 76 images of Bonastent® partially covered biliary stents (Bona PCMS, Sewoon Medical) from 34 patients, were included. In our datasets, all Bona PCMS were inserted endoscopically, whereas the other stent types (Epic, EGIS, Niti-S, Bona UMS) were placed *via* a percutaneous approach. The mean patient age was 75.0 ± 12.2, and 59.8% of the patients were male. Among the 488 images, 169 were radiographs and 319 were fluoroscopic images. This dataset was also divided into 5 sets to institute 5-fold cross-validation, with a training-to-test ratio of approximately 4:1. To enable direct comparison with the primary dataset, the original train/test grouping of the primary dataset was preserved, and only the newly added images were randomly assigned to the five groups. Supplementary Table S1 summarizes the number of images per set, and Table 2 reports the class-wise train/test distribution together with the corresponding patient counts.

**Table 2:** Radiographic or fluoroscopic cases per class and fold, reported as train (patients)/test patients

Class	Fold1 (train/test)	Fold2 (train/test)	Fold3 (train/test)	Fold4 (train/test)	Fold5 (train/test)
Epic	97 (34)/16 (6)	87 (30)/26 (9)	87 (30)/26 (9)	86 (30)/27 (9)	84 (28)/29 (11)
EGIS	84 (30)/37 (14)	101 (36)/20 (8)	90 (34)/31 (11)	104 (38)/17 (6)	96 (35)/25 (10)
NITI-S	133 (48)/37 (14)	120 (45)/50 (17)	134 (50)/36 (12)	122 (45)/48 (17)	140 (52)/30 (10)
Bona UC	44 (19)/7 (3)	35 (15)/16 (7)	41 (17)/10 (5)	46 (20)/5 (2)	40 (18)/11 (4)
Bona PC	59 (27)/17 (7)	59 (27)/17 (7)	61 (27)/15 (7)	60 (27)/16 (7)	61 (27)/15 (7)
Single	344 (134)/88 (35)	332 (130)/100 (39)	340 (135)/92 (34)	355 (140)/77 (29)	338 (134)/94 (35)
Multiple	42 (16)/14 (5)	40 (15)/16 (6)	40 (15)/16 (6)	35 (14)/21 (7)	47 (17)/9 (4)

Numbers in parentheses are the number of patients

### ResNet-50

The ResNet is a widely adopted convolutional neural network backbone in medical image analysis [21]. The ResNet-50 architecture was utilized for end-to-end image recognition and classification. All hyperparameters were manually configured by resizing each input image to 1,536 × 1,536 pixels and applying data augmentation strategies such as mixup [22], cutmix [23], and RandAugment [24] to improve generalization. The detailed training settings are summarized in Supplementary Table S2. Model training was performed on an A100 GPU with 80 GB of memory (NVIDIA).

### Deep learning model development and training

The baseline deep learning model was trained using the primary dataset for three tasks: (1) binary classification to determine whether the image shows a single or multiple biliary stents; (2) four-class classification of stent vendors in cases with a single stent; and (3) binary classification (identification) to determine whether the image contains a specific target stent type. For the augmented dataset, the same three tasks were performed, except that task 2 was extended to a five-class vendor classification by adding the Bona PCMS class.

Both the primary and the augmented datasets were trained from ImageNet [25] pretrained initializations. For adaptation on the augmented dataset, we loaded the task-specific weights obtained on the primary dataset, replaced the vendor classifier with a five-class output layer initialized at random, and then performed full fine-tuning of the entire network. For the identification tasks, we initialized the binary classifier and likewise performed full fine-tuning end-to-end, starting from the primary trained weights.

For adaptation on the augmented dataset, we evaluated three initialization strategies: transfer learning from task-specific weights trained on the primary dataset (Primary init), transfer learning from ImageNet pretrained weights (ImgNet init), and training from random initialization (From Scratch). In the Primary init setting, we loaded the task-specific weights obtained on the primary dataset. For vendor classification, we replaced the vendor classifier with a five-class output layer initialized at random to accommodate the newly introduced Bona PCMS class and then performed end-to-end fine-tuning of the entire network. For the identification tasks, we initialized the binary classifier accordingly and likewise performed end-to-end fine-tuning starting from the primary trained weights. In the ImgNet init setting, we trained the same architectures end-to-end on the augmented dataset, starting from ImageNet pretrained weights without loading primary task weights. In the From Scratch setting, we trained the same architectures end-to-end under the identical protocol with random initialization.

### Generation of class activation map

To assess the interpretability of the ResNet-50 model, class activation maps (CAMs) were generated using gradient-weighted techniques to visualize the discriminative regions that contributed to the final classification outputs. CAMs were produced for both the specific stent type identification and vendor classification tasks. Radiographic input images were passed through trained models, and activation heatmaps were overlaid to reveal spatial areas with high predictive importance.

### Statistical analysis

Categorical and continuous variables were presented as frequencies (*n*) and ratios (%). Model performance was assessed using accuracy and macro-averaged class-wise precision, recall, and F1 scores. Confusion matrices and graphical visualizations were generated using Python (version 3.12.9, Python Software Foundation). All metrics were calculated using the validation folds. Mean values across the cross-validation folds are reported with ± standard deviations. For comparison of receiver operating characteristic (ROC) curves obtained at different fine-tuning epochs during transfer learning, DeLong’s test was performed. Z-statistics from each cross-validation fold were combined using Stouffer’s Z-score method to derive the final *p*-value.

## Results

### ResNet classification performance

For the primary dataset, the ResNet-50 model was trained for 500 epochs, achieving an F1 score of 57.03 ± 6.77 for the task of detecting single *versus* multiple stents. For vendor classification in cases with a single stent, the model attained an F1 score of 94.78 ± 4.07. The identification performance of each stent type, expressed as stent-specific F1 scores, was as follows: Epic™, 97.91 ± 2.59; EGIS, 92.64 ± 4.98; Niti-S, 95.18 ± 1.43; and Bonastent® uncovered, 91.43 ± 3.43.

Using an augmented dataset that incorporated Bonastent® partially covered stents alongside the primary data, we trained the ResNet-50 model for 500 epochs and achieved an F1 score of 60.19 ± 11.71 in detecting single *versus* multiple stents. For vendor classification, the model achieved an F1 score of 92.59 ± 5.81. Stent-specific F1 scores were as follows: Epic™, 98.44 ± 2.16; EGIS, 93.29 ± 5.69; Niti-S, 94.50 ± 0.7; Bonastent® uncovered, 81.52 ± 7.35; and Bonastent® partially covered, 86.32 ± 8.74. To document training dynamics for this setting, we tracked learning curves, and Fig. S3 shows that training and validation losses decreased smoothly without late epoch divergence, while validation F1 and accuracy stabilized after convergence.

**Fig. 1:**
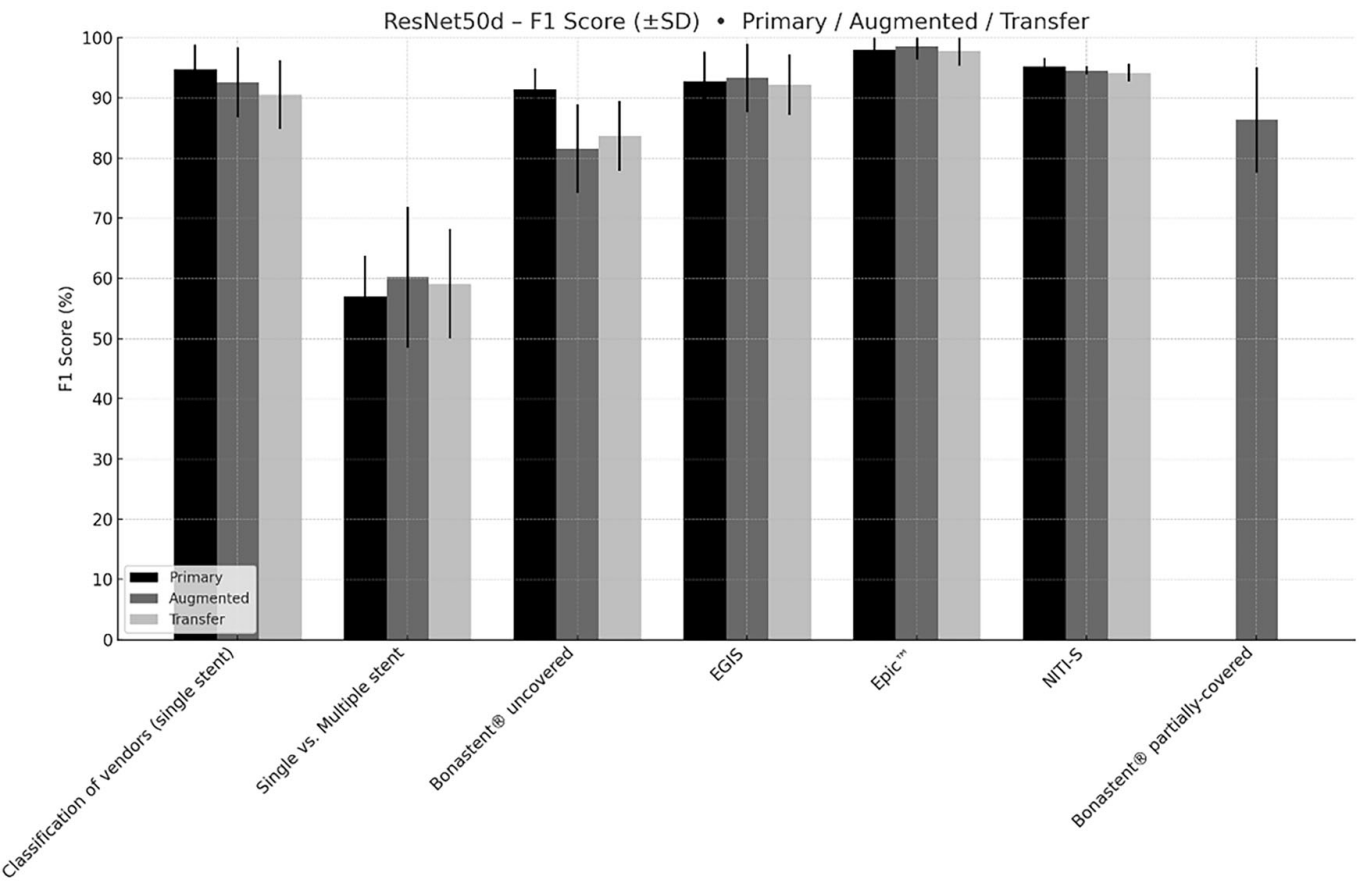
Comparison of F1 scores by dataset and transfer learning. Bar graphs illustrate the F1 scores of the deep learning model across various tasks, comparing performance based on the dataset used (primary *versus* augmented) and transfer learning

After initial training on the primary dataset, the ResNet-50 model underwent transfer learning on the augmented dataset, achieving an F1 score of 59.06 ± 9.08 for single *versus* multiple stent detection. For vendor classification, the model achieved an F1 score of 90.47 ± 5.66. The stent-specific F1 scores were as follows: Epic™, 97.73 ± 2.42; EGIS, 92.15 ± 5.04; Niti-S, 94.09 ± 1.45; and Bonastent® uncovered, 83.58 ± 5.81. Figure 1 presents the comparison of F1 scores by dataset and transfer learning.

To quantify the contribution of transfer learning, we compared three initialization strategies under the same evaluation protocol and summarized the results in Table 3, with the experimental outcomes also visualized in Supplementary Fig. S6. The compared settings were From Scratch with random initialization, transfer learning with ImageNet initialization, and transfer learning with primary dataset initialization. Across all tasks, performance showed a consistent ordering of From Scratch < ImageNet initialization < primary initialization, indicating that pretrained initialization contributes substantially to the achieved performance and that a more domain-relevant initialization yields additional gains. Overall, vendor classification showed consistently high performance for Epic™, Niti-S, and EGIS stents across validation folds, whereas Bonastent® uncovered and partially covered stents accounted for most misclassifications. Misclassification patterns were predominantly observed between the uncovered and partially covered Bonastent® models, reflecting their similar radiographic appearance.

**Table 3:** Accuracy performance of the deep learning model

ResNet	Accuracy	Precision	Recall	F1 score
Single *versus* multiple stent				
Primary dataset	88.92 ± 4.64	84.38 ± 22.95	55.78 ± 3.63	57.03 ± 6.77
Augmented dataset	87.39 ± 4.89	90.91 ± 4.36	59.51 ± 9.93	60.19 ± 11.71
Transfer learning (Primary init)	86.78 ± 4.63	73.81 ± 19.79	58.00 ± 6.41	59.06 ± 9.08
Transfer learning (ImgNet init)	83.68 ± 7.18	71.30 ± 13.19	62.22 ± 6.58	62.41 ± 6.84
From scratch	81.83 ± 12.32	44.69 ± 2.67	49.74 ± 0.58	46.93 ± 1.02
Classification of vendors (single stent)				
Primary dataset	95.42 ± 3.17	95.97 ± 3.09	94.06 ± 4.84	94.78 ± 4.07
Augmented dataset	93.84 ± 4.10	93.32 ± 4.32	92.89 ± 6.41	92.59 ± 5.81
Transfer learning (Primary init)	91.69 ± 4.59	91.85 ± 4.57	90.01 ± 6.45	90.47 ± 5.66
Transfer learning (ImgNet init)	90.07 ± 6.57	89.97 ± 6.41	89.22 ± 7.76	88.58 ± 7.68
From scratch	33.46 ± 2.78	23.38 ± 7.06	26.82 ± 5.55	21.95 ± 5.01
Identification of Epic™				
Primary dataset	98.52 ± 1.69	98.80 ± 1.30	97.23 ± 3.73	97.91 ± 2.59
Augmented dataset	98.92 ± 1.41	99.10 ± 1.00	97.94 ± 3.24	98.44 ± 2.16
Transfer learning (Primary init)	98.37 ± 1.64	98.14 ± 2.22	97.37 ± 2.78	97.73 ± 2.42
Transfer learning (ImgNet init)	97.99 ± 1.50	98.11 ± 1.75	96.12 ± 2.68	97.06 ± 2.24
From scratch	61.23 ± 10.56	53.29 ± 9.25	50.23 ± 3.02	49.21 ± 2.71
Identification of EGIS				
Primary dataset	94.43 ± 4.06	95.64 ± 3.09	91.04 ± 6.60	92.64 ± 4.98
Augmented dataset	95.11 ± 3.68	95.03 ± 2.95	91.70 ± 7.30	93.29 ± 5.69
Transfer learning (Primary init)	94.75 ± 3.30	96.68 ± 2.22	89.50 ± 6.49	92.15 ± 5.04
Transfer learning (ImgNet init)	92.09 ± 3.36	90.69 ± 5.36	87.26 ± 3.40	88.73 ± 4.04
From scratch	71.27 ± 8.82	56.51 ± 1.84	54.12 ± 1.46	53.79 ± 2.37
Identification of NITI-S				
Primary dataset	95.32 ± 1.53	95.53 ± 1.67	95.00 ± 1.18	95.18 ± 1.43
Augmented dataset	94.88 ± 0.76	94.49 ± 1.24	94.61 ± 0.62	94.50 ± 0.70
Transfer learning (Primary init)	94.51 ± 1.59	94.27 ± 1.41	94.01 ± 1.71	94.09 ± 1.45
Transfer learning (ImgNet init)	92.97 ± 3.67	93.65 ± 3.75	91.59 ± 4.50	92.32 ± 4.07
From scratch	56.19 ± 9.05	55.14 ± 6.30	54.09 ± 5.06	52.48 ± 4.89
Identification of Bonastent® uncovered				
Primary dataset	96.95 ± 1.81	98.34 ± 1.03	86.81 ± 4.50	91.43 ± 3.43
Augmented dataset	94.44 ± 2.86	87.45 ± 6.87	77.81 ± 7.60	81.52 ± 7.35
Transfer learning (Primary init)	96.28 ± 1.04	94.72 ± 3.48	78.41 ± 8.38	83.58 ± 5.81
Transfer learning (ImgNet init)	93.32 ± 2.98	86.66 ± 7.37	71.70 ± 10.31	74.84 ± 6.84
From scratch	82.29 ± 9.91	54.61 ± 4.85	59.75 ± 9.71	54.52 ± 5.32
Identification of Bonastent® partially covered				
Primary dataset	N/A	N/A	N/A	N/A
Augmented dataset	94.02 ± 3.27	92.52 ± 5.96	82.71 ± 9.63	86.32 ± 8.74
Transfer learning	N/A	N/A	N/A	N/A

Numbers in each cells means average of each group in the dataset with standard deviation in parentheses. Model training on both the primary and augmented datasets was conducted for 500 epochs. Transfer learning (ImgNet init and Primary init) and From Scratch (random initialization) were all trained for 100 epochs using identical training hyperparameters and protocol, with initialization being the only difference. The performance of Bonastent® partially covered stent identification was not evaluated, as this stent type was not included in the primary dataset. Transfer learning cannot be performed without a model trained on the primary dataset

**Fig. 2:**
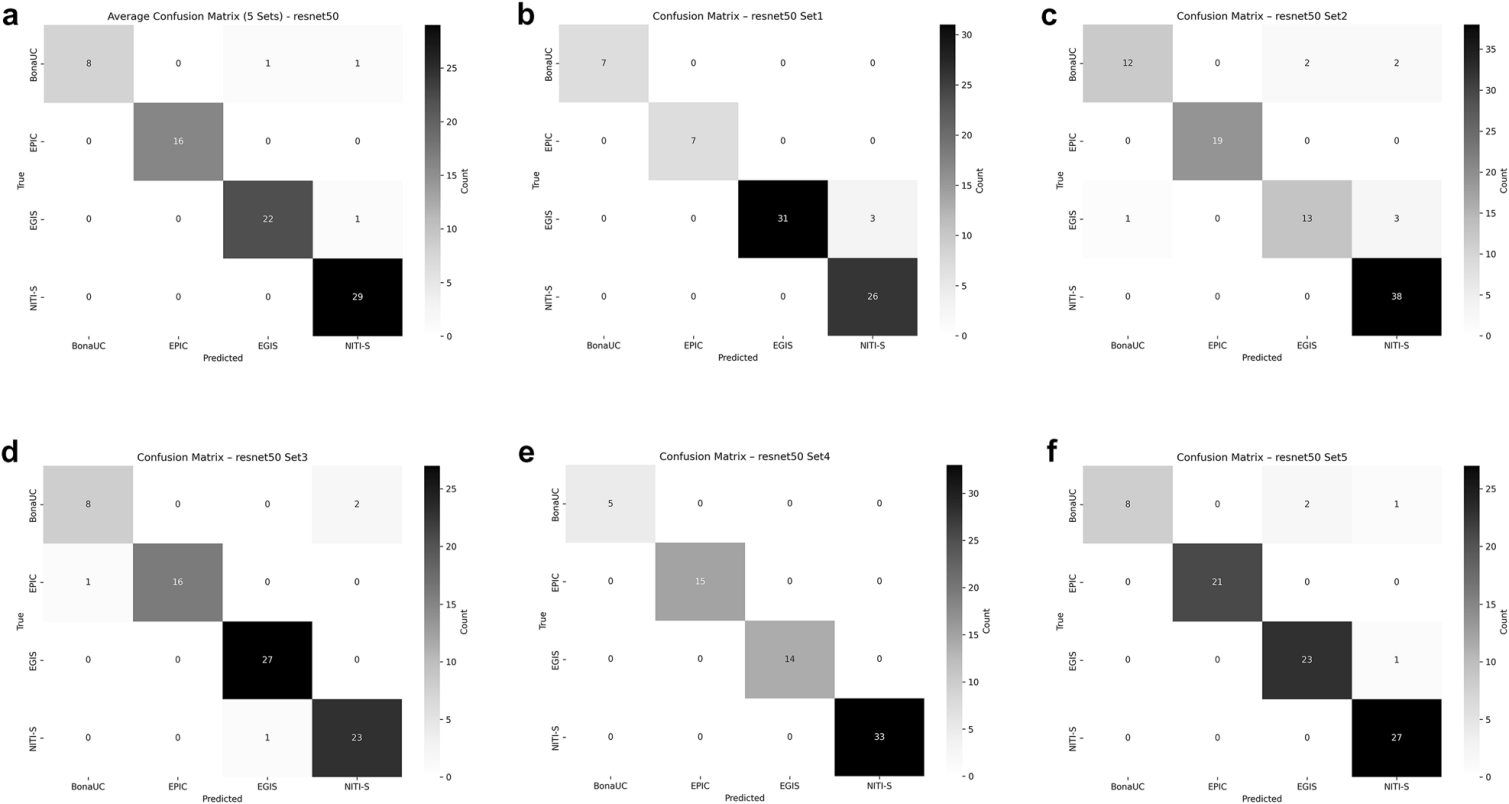
Confusion matrices for vendor classification by the deep learning model in the case of a single stent across five validation sets and their average. Each matrix corresponds to one of the five validation sets, with the average matrix shown alongside: **a** average; **b–f** Set 1-5

The performance for the classification of the Bonastent® partially covered stent was not evaluated in the primary dataset, as this stent type was absent. Consequently, transfer learning could not be performed. Table 3 presents the detailed performance achieved by the ResNet model. Figure 2 presents the confusion matrices of the classification of vendors in case of a single stent across five validation sets and their average. Additionally, the experimental results for EfficientNet-B0 and DeiT are summarized in Supplementary Table S3 and Supplementary Figs. S1 and S2.

We also examined whether performance varied across acquisition settings by stratifying all held-out test results by acquisition type (fluoroscopic x-ray *versus* computed radiography) and comparing class-specific classification performance within each stratum. Overall, we observed only small differences between fluoroscopic x-ray and computed radiography across tasks and classes (Supplementary Fig. S4).

To assess whether class imbalance materially affected performance, we conducted a sensitivity comparison between standard cross entropy and focal loss under the same evaluation protocol (Supplementary Fig. S5). Across folds, focal loss produced performance comparable to or slightly lower than cross entropy in terms of macro Precision, macro Recall, and macro F1, suggesting that imbalance was not a dominant limiting factor in this setting.

### ROC analysis

**Fig. 3:**
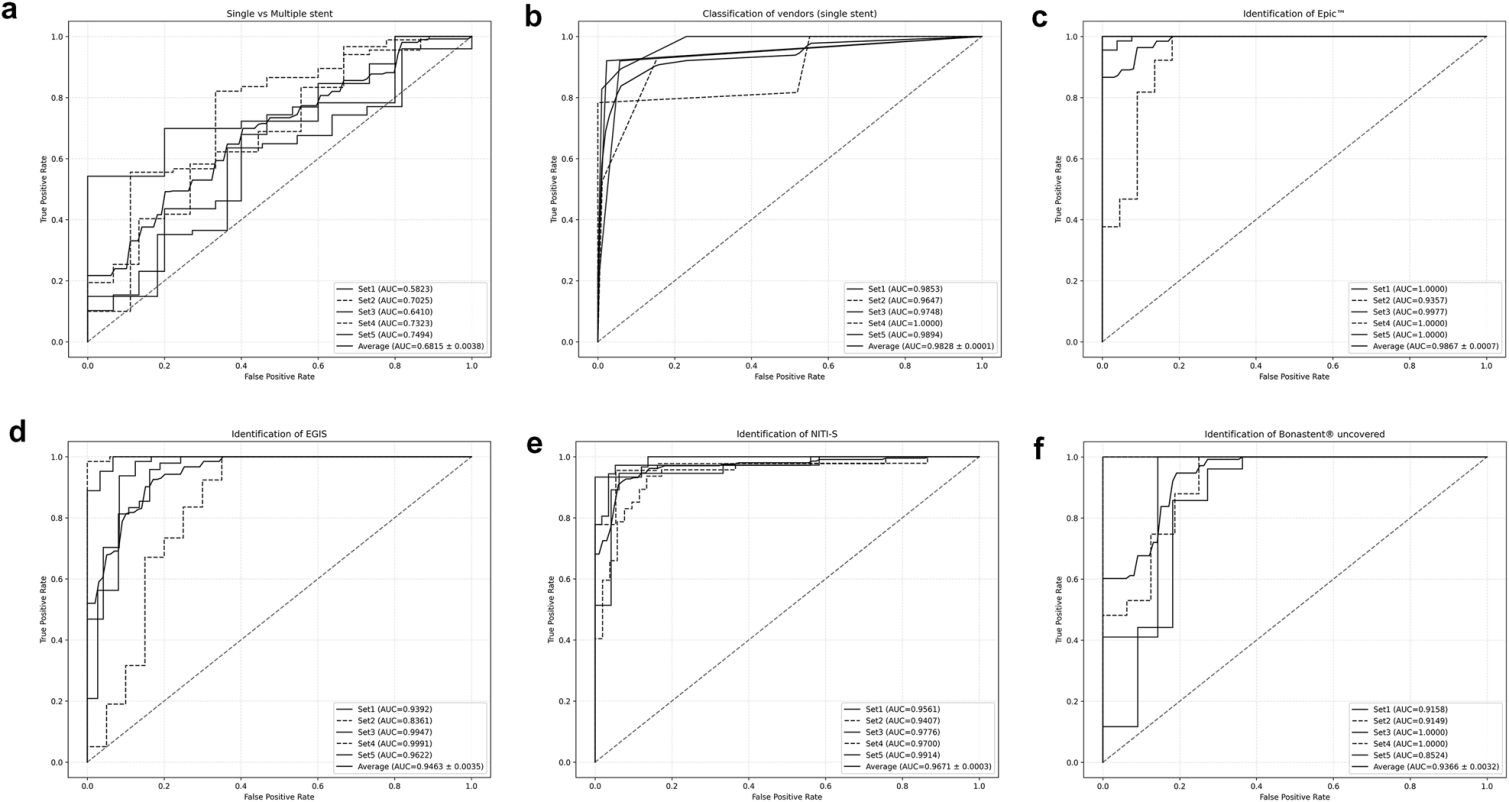
ROC curves for biliary stent classification for six primary classification tasks: (**a**) single *versus* multiple stents; (**b**) vendor classification; (**c**) EPIC; (**d**) EGIS; (**e**) NITI-S; and (**f**) Bona uncovered stent identification. Each plot includes five validation sets with the averaged curve in gray

**Fig. 4:**
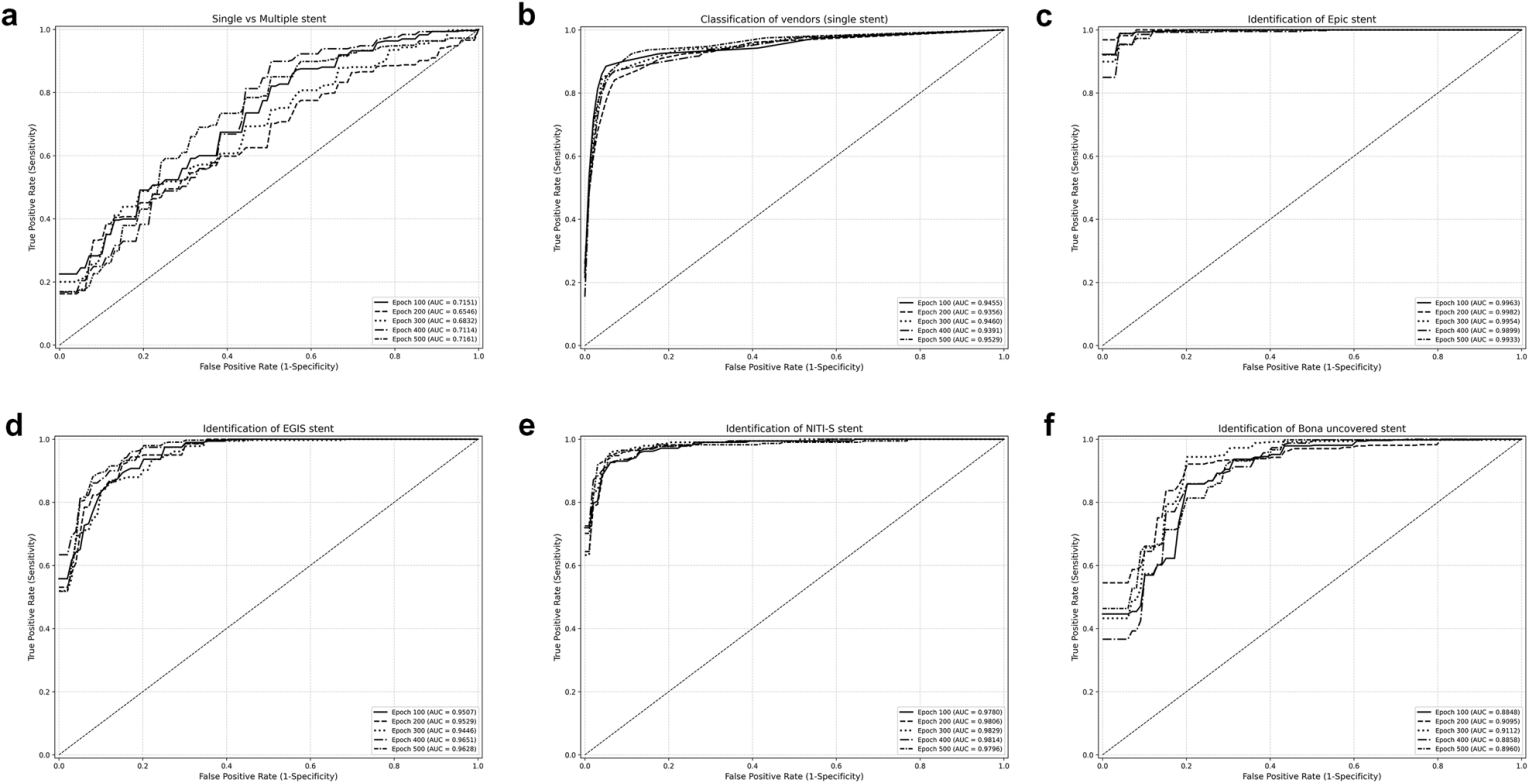
Receiver operating characteristic curves comparing different transfer learning epochs across six biliary stent classification tasks. Each curve represents the mean performance across five validation folds for a given training epoch: (**a**) Single *versus* Multiple stent; (**b**) Classification of vendors (single stent); (**c**) Identification of Epic™; (**d**) Identification of EGIS; (**e**) Identification of NITI-S; (**f**) Identification of Bonastent® uncovered

**Fig. 5:**
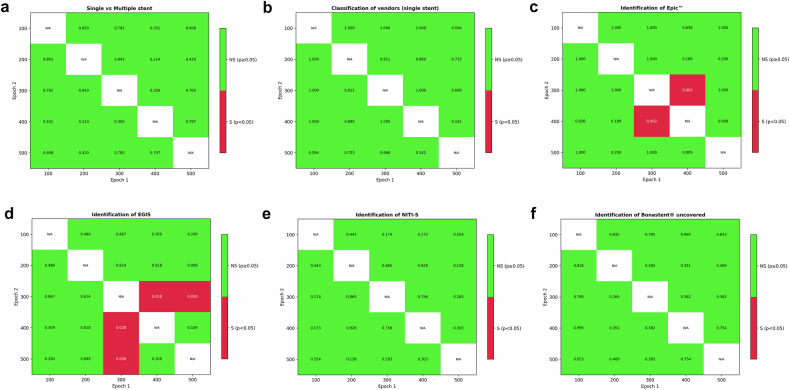
Statistical assessment of epoch-wise model similarity for transfer learning. DeLong’s test showed that most receiver operating characteristic curve comparisons between fine-tuning epochs during transfer learning were not statistically significant (green color); however, several epoch pairs demonstrated significant differences (red color): (**a**) Single *versus* Multiple stent; (**b**) Classification of vendors (single stent); (**c**) Identification of Epic™; (**d**) Identification of EGIS; (**e**) Identification of NITI-S; (**f**) Identification of Bonastent® uncovered

ROC analysis was performed for all primary biliary stent classification tasks. For each task, ROC curves were constructed by applying conditional score correction: the original prediction score was used when the predicted class matched the target class, and 1 minus the score otherwise. Table 4 shows the performance of each classification task based on ROC analysis. Specifically, the classification of single *versus* multiple stents achieved an average area under the ROC curve (AUROC) of 0.6815 ± 0.0038 across five validation sets. For vendor classification among single stent cases, the average AUROCs for individual vendors were as follows: Epic™ (1.0000 ± 0.0000), Niti S (0.9878 ± 0.0108), Bonastent® uncovered (0.9706 ± 0.0313), and EGIS (0.9729 ± 0.0373). The mean macro AUROC across folds was 0.9828 ± 0.0121 with a 95% bootstrap confidence interval of 0.9711 to 0.9923. Because the number of validation samples per vendor class was limited, uncertainty was estimated using bootstrap resampling of the held-out validation images, and foldwise 95% bootstrap confidence intervals are reported alongside the per vendor AUROCs. Epic™ achieved an AUROC of 1.00 with zero variance because all Epic cases were correctly classified in every held-out fold, and such very high AUROC values should be interpreted with caution in small sample settings. Figure 3 presents the ROC curves for all primary stent classification models, including per-set curves and the averaged curve for each task. Figure 4 presents the comparison of different transfer learning epochs for biliary stent classification using ROC curves. DeLong’s test showed that most ROC curve comparisons between fine-tuning epochs during transfer learning were not statistically significant; however, several epoch pairs demonstrated significant differences, particularly in the identification of EGIS and Epic™ stents (Fig. 5).

**Table 4:** Performance of stent classification tasks measured by AUROC

Classification task	AUROC (mean ± standard deviation)
Single *versus* multiple stents	0.6815 ± 0.0038
Classification of vendors	0.9828 ± 0.0001 (average) [0.9711, 0.9923]
EPIC	1.00 ± 0 [1.0000, 1.0000]
EGIS	0.9729 ± 0.0373 [0.7953, 1.0000]
NITI-S	0.9878 ± 0.0108 [0.9295, 1.0000]
Bona uncovered	0.9706 ± 0.0313 [0.7802, 1.0000]
Identification of EPIC	0.9867 ± 0.007
Identification of EGIS	0.9463 ± 0.0035
Identification of NITI-S	0.9671 ± 0.0003
Identification of Bona uncovered	0.9366 ± 0.0032

For the vendor classification task only, 95% confidence intervals were estimated by nonparametric bootstrap resampling of the held-out validation images within each fold (2,000 resamples). Given the limited number of samples per vendor, the reported CI summarizes the foldwise bootstrap intervals

### Visualization of the class activation map

**Fig. 6:**
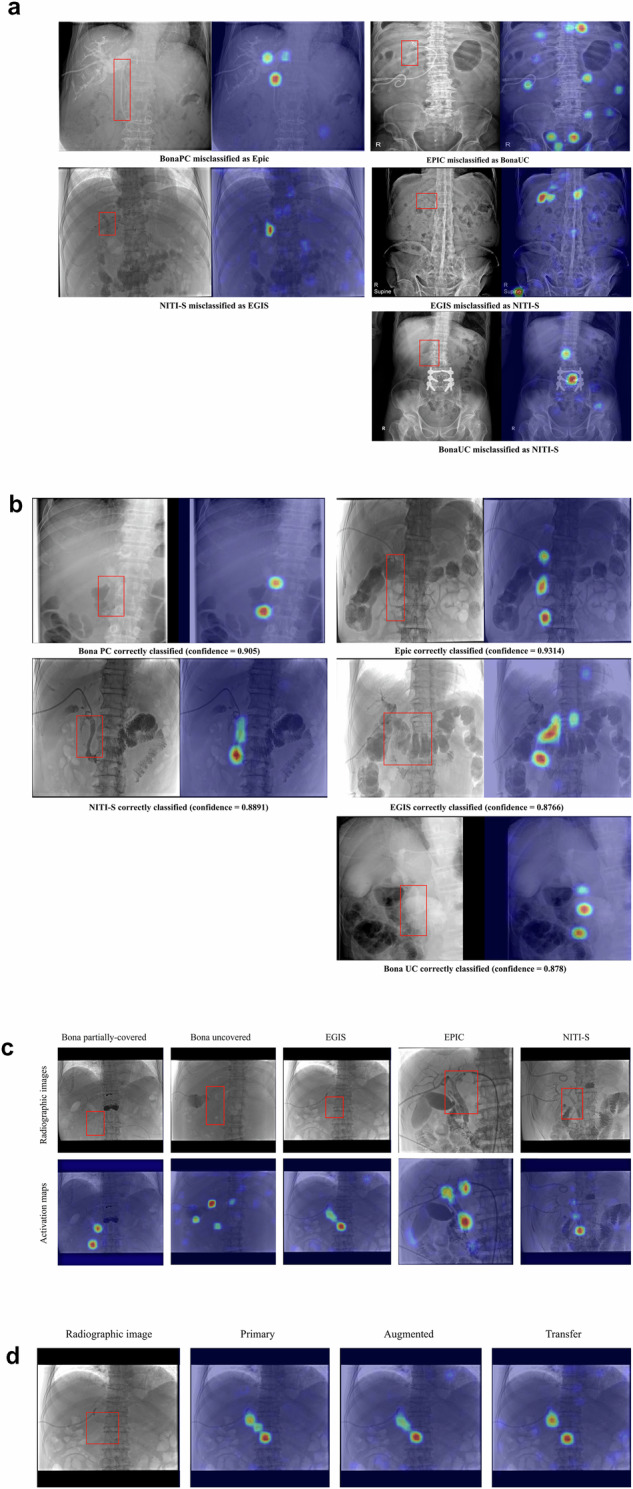
Visualization of class activation maps. Examples of class activation maps highlighting discriminative regions used for stent classification: (**a**) vendor classification; (**b**) comparison across different datasets; (**c**) augmented dataset misclassification examples; (**d**) augmented dataset cases correctly classified with high confidence. Numbers in each cells means average of each group in the dataset. Parentheses means standard deviation. Model training on both the primary and augmented datasets was conducted for 500 epochs. Transfer learning was performed over 100 epochs. The performance of Bonastent® partially covered stent identification was not evaluated, as this stent type was not included in the primary dataset. Transfer learning cannot be performed without a model trained on the primary dataset

In the vendor classification task, CAMs highlighted salient regions that spatially corresponded with the stent structures (Fig. 6). For clarity, the stent region in Fig. 6 is delineated by a red bounding box. Figure 6a shows misclassified examples in which activation is spatially diffuse and often outside the boxed stent region. By contrast, Fig. 6b shows high-confidence correct cases, where activation is concentrated within the stent box and aligns with stent-specific morphological features such as the wire mesh, radiopaque markers. These observations indicate that correct decisions are associated with attention to clinically relevant stent features while errors arise when the network relies on spurious context. This additional analysis strengthens the interpretability of the ResNet-based model and supports the view that the highlighted regions correspond to the stent rather than to unrelated background cues.

Figure 6c shows that the most activated regions, typically represented in yellow to red, were primarily localized along the stent margins, radiopaque markers, and wire mesh patterns—demonstrating that the model relied on relevant morphological cues for prediction. For the training strategy comparison (Fig. 6d), even when fine-tuned *via* transfer learning for only 20% of the augmentation epochs, model Grad CAM activations around the stent remained virtually unchanged. These activations were nearly identical to those observed in models trained with the full augmentation schedule or with the primary dataset for all epochs. In each scenario, activations were tightly localized to the stent with minimal background noise. Representative Grad CAM maps in Fig. 6 illustrate that only a fraction of the transfer learning epochs is necessary to achieve stent localization performance comparable to full training, thereby reducing computational cost without compromising interpretability.

## Discussion

The main findings of this study demonstrated that the DL model (ResNet-50) effectively detects and classifies biliary stents from radiographic and fluoroscopic images. Specifically, the ResNet-50 model achieved reliable performance in distinguishing single *versus* multiple stents, classifying stent vendors, and identifying individual stent types. Additionally, transfer learning methods efficiently integrated newly introduced stent designs without compromising classification performance. The ResNet model in routine practice may improve procedural planning in reintervention.

To the best of our knowledge, this is the first study to evaluate a DL model for biliary stent classification using radiographic and fluoroscopic images. Although direct comparisons are not possible due to the absence of prior research, similar applications of DL have been reported in the context of other stents. For instance, previous studies by Kappe et al and Breininger et al demonstrated the feasibility of automated stent-graft segmentation during endovascular aortic repair procedures using digital subtraction angiography and fluoroscopic images, respectively [19, 20]. These studies support the applicability of CNN-based methods for automated recognition and classification tasks involving metallic implantable devices, highlighting the potential clinical value of our results in biliary stent management.

In this study, the ResNet-50 model performance in distinguishing single *versus* multiple biliary stents was relatively low, with an average F1 score of 57.03 ± 6.77 in the baseline dataset, compared to the reliable performance in vendor classification tasks, where F1 scores ranged from 91.43 ± 3.43 to 97.91 ± 2.59. A possible explanation for this discrepancy is that single vendor classification primarily relies on distinct structural patterns and localized visual features of individual stents, such as mesh configurations and radiopaque markers, which the ResNet-50 model can easily learn and differentiate. Conversely, identifying single *versus* multiple stents demands that the ResNet-50 model captures more global and spatially complex visual patterns, particularly involving the recognition of spatial relationships and overlaps among multiple stents. The similarity in visual characteristics between overlapping stents likely hindered accurate feature extraction by the ResNet, reducing classification accuracy. Additionally, the global complexity of identifying multiple stents compared to localized vendor-specific features may have further contributed to decreased performance. Further studies using larger and balanced datasets, possibly combined with specialized network architectures optimized for spatial relationship recognition, may improve the accuracy of single *versus* multiple stent identification.

The transfer learning results suggest that initialization is an important factor for achieving strong performance in our setting, where labeled data are limited. The consistent ordering observed in Table 3 indicates that pretrained initialization tends to outperform random initialization, and the additional gain with primary dataset initialization over ImageNet initialization is in line with the potential value of domain-relevant pretraining for fluoroscopic stent classification. From a practical perspective, we evaluated fine-tuning schedules from 100 to 500 epochs and did not observe statistically significant differences across epoch settings based on the DeLong test, which supports using 100 epochs, corresponding to 20% of the 500-epoch budget used for full augmented training, as a reasonable training budget in our experiments. Collectively, these findings suggest a resource-efficient update pathway in which newly introduced stent designs may be incorporated through targeted fine-tuning rather than full retraining, potentially making iterative model maintenance more feasible in real-world deployments. However, a practical limitation was noted when applying the current transfer learning approach. Specifically, transfer learning requires an initial baseline model trained on datasets containing visual features representative of subsequently introduced classes. Since the Bonastent® partially covered stent was not included in our primary dataset, loading the weights from the model trained on the primary dataset was not possible for this particular stent. Consequently, stent-specific performance evaluation could not be conducted for this stent type. To overcome this limitation, future baseline datasets should comprehensively include a broader range of existing and anticipated stent types. Capturing and incorporating *ex vivo* images of biliary stents from multiple angles could further enhance the model’s ability to generalize and recognize novel designs in clinical imaging. Additionally, data augmentation techniques might be employed to facilitate adaptation to previously unseen stent types.

This study has several limitations. First, the dataset was relatively small and derived from a single center, potentially limiting the generalizability of the results. Although internal validation was performed using cross-validation, external evaluation was not performed because stent vendors and device models differ across institutions, making it difficult to obtain an external dataset whose class composition aligns with the label space used in this study. Additionally, the dataset contained heterogeneous numbers of radiographic or fluoroscopic images across different stent types, possibly introducing classification biases. While stent types were verified using objective device codes to minimize subjective bias, the reliance on a single annotator precludes the assessment of inter-rater or intra-rater variability. Even with procedural records, the possibility of clerical errors during image selection cannot be completely ruled out. To address this, future studies should employ a multi-reader design with independent validation or consensus labeling to ensure the robustness of the ground truth. Furthermore, such work should expand and explore the integration of these automated stent recognition systems into routine fluoroscopic workflows for procedural support.

In conclusion, the ResNet-50 model demonstrated reliable performance in classifying biliary stents on radiographic and fluoroscopic images. Transfer learning allowed the model to be updated to incorporate newly introduced stent types with minimal degradation in performance. These results indicate the potential applicability of model-updating strategies for device-related image analysis. Further validation with larger and multicenter datasets will be necessary to establish their clinical utility.

## Supplementary information

**Table S1** Number of radiographic or fluoroscopic images for primary or augmented dataset. **Table S2** Model training settings. **Table S3** Accuracy performance of EfficientNet b0, DeiT. **Fig. S1** Comparison of F1 scores by dataset and transfer learning(EfficientNet B0, DeiT). Bar graphs illustrate the F1 scores of the EfficientNetB0, DeiT across various tasks, comparing performance based on the dataset used (primary vs. augmented) and transfer learning. **Fig. S2** Confusion matrices of classification of vendors in case of single stent average(EfficnetNetB0, DeiT). Average confusion matrices for EfficientNet-B0 and DeiT, summarizing vendor classification in single-stent cases. **Fig. S3** Learning curves across training for the vendor classification model on the augmented dataset using five fold cross-validation: (**a**) Training loss over epochs; (**b**) Validation loss over epochs; (**c**) Validation accuracy over epochs; (**d**) Validation F1 score over epochs. **Fig. S4** Stratified analysis of model performance by acquisition modality (fluoroscopic x-ray *versus* computed radiography): (**a**) Single versus multiple stents; (**b**) Vendor classification; (**c**) Epic™ identification; (**d**) EGIS identification. (**e**) Niti S identification; (**f**) Bonastent® uncovered identification; (**g**) Bonastent® partially covered identification. **Fig. S5** Focal loss ablation for vendor classification on the augmented dataset. Comparison of vendor classification performance trained with and without focal loss under identical hyperparameters for 500 epochs using five fold cross-validation. **Fig. S6** Comparison of initialization strategies across tasks**:** (**a**) Macro F1 score. (**b**) Accuracy. (**c**) Macro precision. (**d**) Macro recall. Scores are reported as mean ± standard deviation across five sets for each task under three initialization strategies: From Scratch (random initialization), Transfer learning (ImgNet init), and Transfer learning (Primary init).

## Data Availability

The datasets generated or analyzed during the current study are available from the corresponding author (KY Kim, M.D., Ph.D.) upon reasonable request.

## Abbreviations

AUROC: Area under the receiver operating characteristic curve
CAM: Class activation map
CNN: Convolutional neural network
DL: Deep learning
ROC: Receiver operating characteristic
SEMS: Self-expandable metallic stent
