# Supplementary material for: Deep learning-based biliary stent classification and transfer learning adaptation to an additional stent type
Source: Eur Radiol Exp. 2026 Jun 8;10:85. doi: 10.1186/s41747-026-00749-4 (PMC13247018; doi:10.1186/s41747-026-00749-4)
Supplement: Supplementary file 1 — Table S1 Number of radiographic or fluoroscopic images for primary or augmented dataset. Table S2 Model training settings. Table S3 Accuracy performance of EfficientNet b0, DeiT. Fig. S1 Comparison of F1 scores by dataset and transfer learning(EfficientNet B0, DeiT). Bar graphs illustrate the F1 scores of the EfficientNetB0, DeiT across various tasks, comparing performance based on the dataset used (primary vs. augmented) and transfer learning. Fig. S2 Confusion matrices of classification of vendors in case of single stent average(EfficnetNetB0, DeiT). Average confusion matrices for EfficientNet-B0 and DeiT, summarizing vendor classification in single-stent cases. Fig. S3 Learning curves across training for the vendor classification model on the augmented dataset using five fold cross-validation: (a) Training loss over epochs; (b) Validation loss over epochs; (c) Validation accuracy over epochs; (d) Validation F1 score over epochs. Fig. S4 Stratified analysis of model performance by acquisition modality (fluoroscopic x-ray versus computed radiography): (a) Single versus multiple stents; (b) Vendor classification; (c) Epic™ identification; (d) EGIS identification. (e) Niti S identification; (f) Bonastent® uncovered identification; (g) Bonastent® partially covered identification. Fig. S5 Focal loss ablation for vendor classification on the augmented dataset. Comparison of vendor classification performance trained with and without focal loss under identical hyperparameters for 500 epochs using five fold cross-validation. Fig. S6 Comparison of initialization strategies across tasks: (a) Macro F1 score. (b) Accuracy. (c) Macro precision. (d) Macro recall. Scores are reported as mean ± standard deviation across five sets for each task under three initialization strategies: From Scratch (random initialization), Transfer learning (ImgNet init), and Transfer learning (Primary init). [file 41747_2026_749_MOESM1_ESM.pdf]

Deep learning based biliary stent classification and transfer learning adaptation to an additional stent type

ELECTRONIC SUPPLEMENTARY MATERIAL

For reproducibility we have released the deep learning training code together with the statistical analysis and visualization scripts. The repository is available at: https://github.com/AISeedHub/Deep-learning-based-biliary-stent-classification

Table S1 Number of radiographic or fluoroscopic images for primary or augmented dataset.

Identification of stents	Primary set			Augmented set		
	Train	Test	Total	Train	Test	Total
Set 1	327	85	412	386	102	488
Set 2	313	99	412	372	116	488
Set 3	319	93	412	380	108	488
Set 4	330	82	412	390	98	488
Set 5	324	88	412	385	103	488
**Classification of vendors**	**Primary set**			**Augmented set**		
	**Train**	**Test**	**Total**	**Train**	**Test**	**Total**
Set 1	296	74	370	344	88	432
Set 2	280	90	370	332	100	432
Set 3	292	78	370	340	92	432
Set 4	303	67	370	355	77	432
Set 5	287	83	370	338	94	432

Table S2 Model training settings

**Training setting**	**Configuration**
Image resolution	(1536,1536)
Batch size	32
Optimizer	AdamW
Learning rate	1e-3
Weight decay	5e-2
Random Erasing	0.25
Warmup epochs	10
Training epochs	500(Primary, Augmented), 100~500(Transfer learning)
Augmentation	RandAug
Label smoothing	0.1
Mixup	1.0
Cutmix	0.8

Table S3 Accuracy performance of EfficientNet b0, DeiT

Effici0entNet B0	Accuracy	Precision	Recall	F1 score
Single vs. Multiple stent				
Primary dataset	90.33 ± 3.97	90.35 ± 8.77	64.03 ± 8.21	67.53 ± 10.24
Augmented dataset	87.58 ± 4.54	80.47 ± 22.18	58.92 ± 6.11	60.87 ± 10.00
Transfer learning(Primary init)	86.61 ± 3.96	69.96 ± 24.25	53.97 ± 4.01	53.34 ± 6.72
Classification of vendors (single stent)				
Primary dataset	94.63 ± 3.59	95.09±3.80	93.86 ± 3.61	94.25 ± 3.60
Augmented dataset	94.32 ± 2.95	93.96 ± 3.62	94.35 ± 3.63	94.42 ± 3.09
Transfer learning(Primary init)	93.80 ± 3.32	94.55 ± 3.01	93.23 ± 4.96	93.66 ± 4.15
Identification of Epic™				
Primary dataset	99.60 ± 0.90	99.75 ± 0.57	99.09 ± 2.03	99.40 ± 1.35
Augmented dataset	99.31 ± 1.54	99.26 ± 1.66	98.74 ± 2.83	98.98 ± 2.28

Transfer learning(Primary init)	98.91 ± 1.41	98.99 ± 1.55	98.02 ± 2.59	98.48 ± 2.10
Identification of EGIS				
Primary dataset	94.01 ± 4.13	94.37 ± 3.33	90.98 ± 6.45	92.13 ± 5.20
Augmented dataset	95.68 ± 3.25	95.74 ± 3.56	92.80 ± 5.71	93.99 ± 4.33
Transfer learning(Primary init)	94.19 ± 4.11	93.02 ± 4.70	91.01 ± 7.10	91.85 ± 5.97
Identification of NITI-S				
Primary dataset	96.59 ± 2.58	96.45 ± 2.59	96.76 ± 2.60	96.53 ± 2.59
Augmented dataset	97.10 ± 2.21	96.58 ± 2.19	96.10 ± 1.68	96.25 ± 1.91
Transfer learning(Primary init)	95.04 ± 1.69	94.84 ± 1.48	94.60 ± 1.68	94.69 ± 1.51
Identification of Bonastent® uncovered				
Primary dataset	97.15 ± 1.09	96.52 ± 2.98	88.72 ± 2.96	91.98 ± 1.43
Augmented dataset	95.74 ± 2.21	94.70±3.99	80.24 ± 6.30	85.07 ± 4.12
Transfer learning(Primary init)	92.67 ± 2.13	73.65±15.17	68.28 ± 11.45	68.94 ± 11.70

Identification of Bonastent® partially-covered				
Primary dataset	N/A	N/A	N/A	N/A
Augmented dataset	95.45 ± 2.27	94.63 ± 4.13	87.17 ± 5.99	90.29 ± 5.13
**DeiT**	**Accuracy**	**Precision**	**Recall**	**F1 score**
Single vs. Multiple stent				
Primary dataset	90.31 ± 4.24	55.14 ± 20.88	50.67 ± 1.49	48.67 ± 2.10
Augmented dataset	85.50 ± 4.54	42.75 ± 2.27	50.00 ± 0.00	46.07 ± 1.33
Transfer learning(Primary init)	85.88 ± 4.85	59.67 ± 24.90	52.74 ± 4.59	50.67 ± 8.35
Classification of vendors (single stent)				
Primary dataset	90.37 ± 4.92	91.20 ± 4.70	89.21 ± 5.46	89.87 ± 5.27
Augmented dataset	83.10 ± 5.45	81.53 ± 6.79	78.56 ± 6.28	79.31 ± 6.20
Transfer learning(Primary init)	80.35 ± 8.29	72.69 ± 12.03	73.39 ± 9.59	71.86 ± 10.67

Identification of Epic™				
Primary dataset	91.31 ± 4.88	93.17 ± 3.81	83.29 ± 8.72	86.32 ± 7.45
Augmented dataset	87.76 ± 8.95	86.14 ± 14.17	76.73 ± 14.75	78.17 ± 18.65
Transfer learning(Primary init)	90.37 ± 4.72	92.04 ± 4.16	81.34 ± 7.24	84.78 ± 6.38
Identification of EGIS				
Primary dataset	89.13 ± 4.54	92.70 ± 3.95	78.95 ± 5.56	82.86 ± 5.72
Augmented dataset	84.10 ± 6.12	81.02 ± 5.33	74.20 ± 5.76	76.14 ± 6.12
Transfer learning(Primary init)	79.63 ± 6.41	77.65 ± 3.31	61.42 ± 6.93	61.93 ± 9.17
Identification of NITI-S				
Primary dataset	90.04 ± 3.00	89.61 ± 3.12	90.47 ± 2.51	89.81 ± 2.89
Augmented dataset	84.79 ± 6.76	86.90 ± 2.39	85.80 ± 3.10	86.18 ± 2.84
Transfer learning(Primary init)	87.51 ± 2.64	86.92 ± 2.13	86.61 ± 2.51	86.53 ± 2.19
Identification of Bonastent® uncovered				

Primary dataset	96.75 ± 2.12	97.00 ± 3.72	87.20 ± 4.31	91.23 ± 3.62
Augmented dataset	93.93 ± 1.52	89.42±5.64	70.52 ± 3.91	75.97 ± 3.51
Transfer learning(Primary init)	92.35 ± 2.70	80.96±19.70	59.53 ± 6.81	62.52 ± 9.50
Identification of Bonastent® partially-covered				
Primary dataset	N/A	N/A	N/A	N/A
Augmented dataset	88.25 ± 1.17	87.63 ± 7.90	64.27 ± 7.97	67.11 ± 7.95

Numbers in each cells means average of each groups in dataset. Parentheses means standard deviation.

Model training on both the primary and augmented datasets was conducted for 500 epochs.Transfer learning was performed over 100 epochs. The performance of Bonastent® partially-covered stent identification was not evaluated, as this stent type was not included in the primary dataset. Transfer learning cannot be performed without a model trained on the primary dataset.

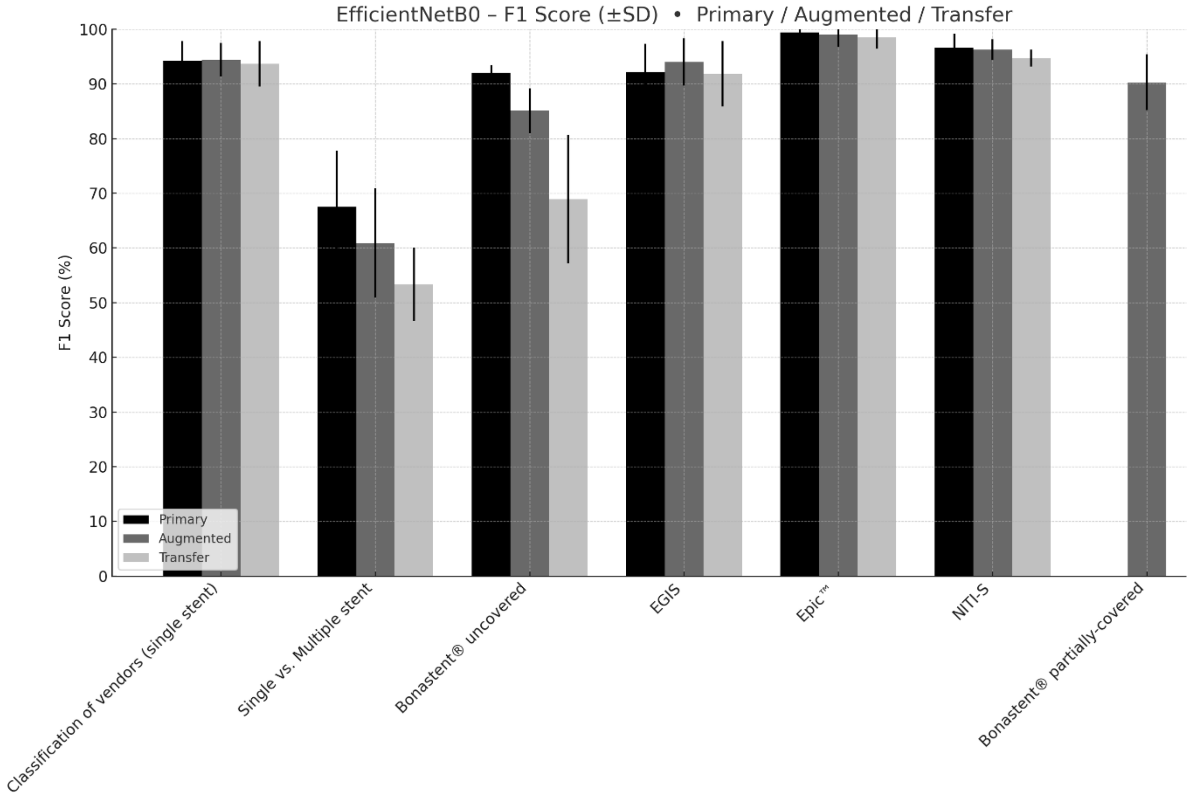
EfficientNetB0 – F1 Score (±SD) • Primary / Augmented / Transfer
F1 Score (%)
Primary
Augmented
Transfer
Classification of vendors (single stent)
Single vs. Multiple stent
Bonastent® uncovered
EGIS
Epic™
NITI-S
Bonastent® partially-covered

(a)

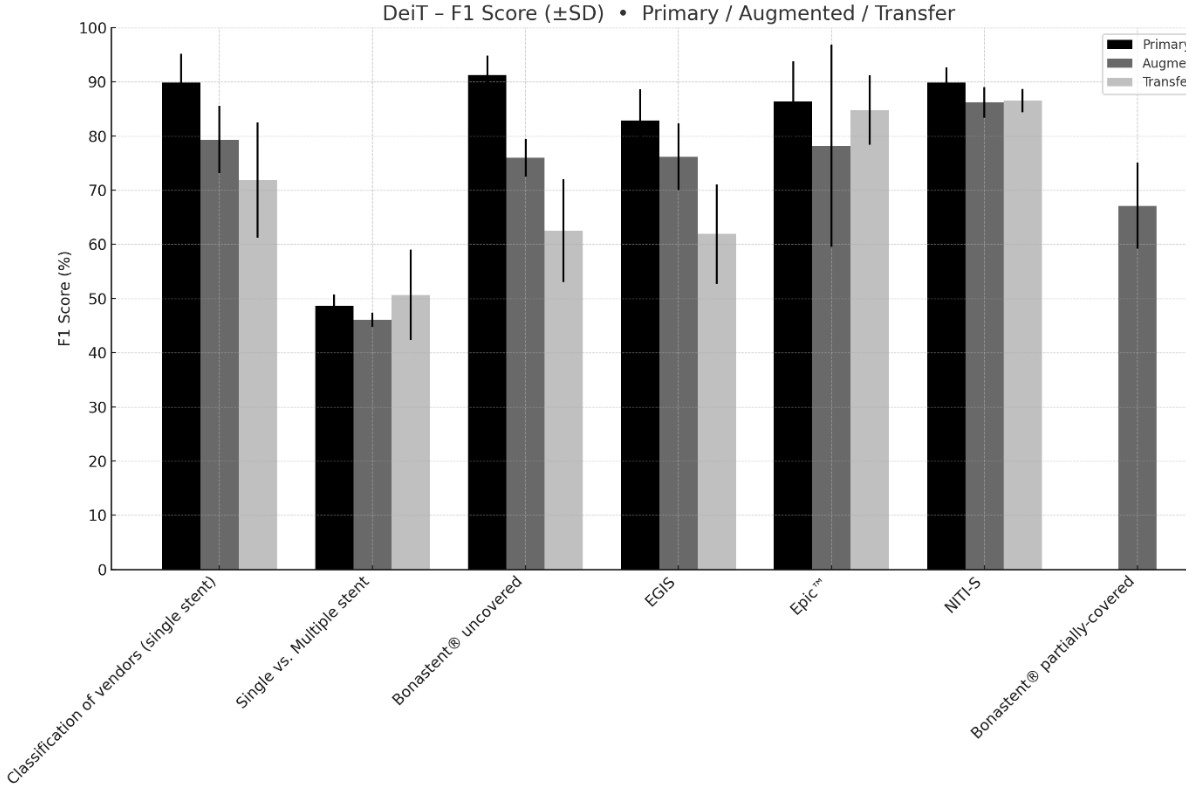
DeiT – F1 Score (±SD) • Primary / Augmented / Transfer
F1 Score (%)
Classification of vendors (single stent)
Single vs. Multiple stent
Bonastent® uncovered
EGIS
Epic™
NITI-S
Bonastent® partially-covered

(b)

Fig. S1 Comparison of F1 scores by dataset and transfer learning(EfficientNet B0, DeiT). Bar graphs illustrate the F1 scores of the EfficientNetB0, DeiT across various tasks, comparing performance based on the dataset used (primary vs. augmented) and transfer learning.

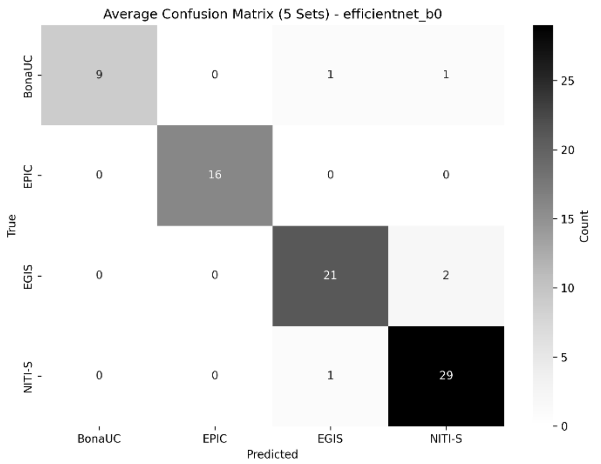

(a)

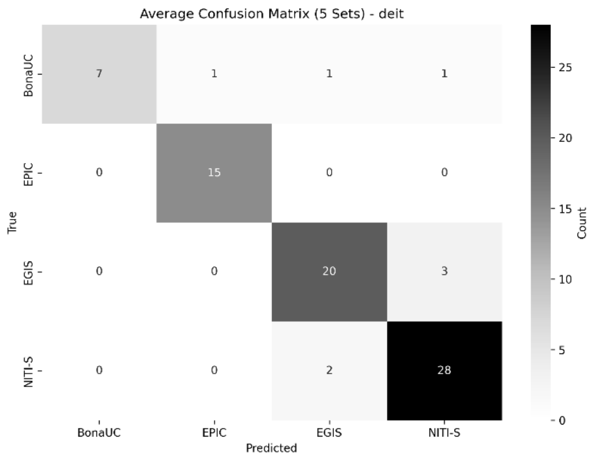

(b)

Fig. S2 Confusion matrices of classification of vendors in case of single stent average(EfficnetNetB0, DeiT). Average confusion matrices for EfficientNet-B0 and DeiT, summarizing vendor classification in single-stent cases.

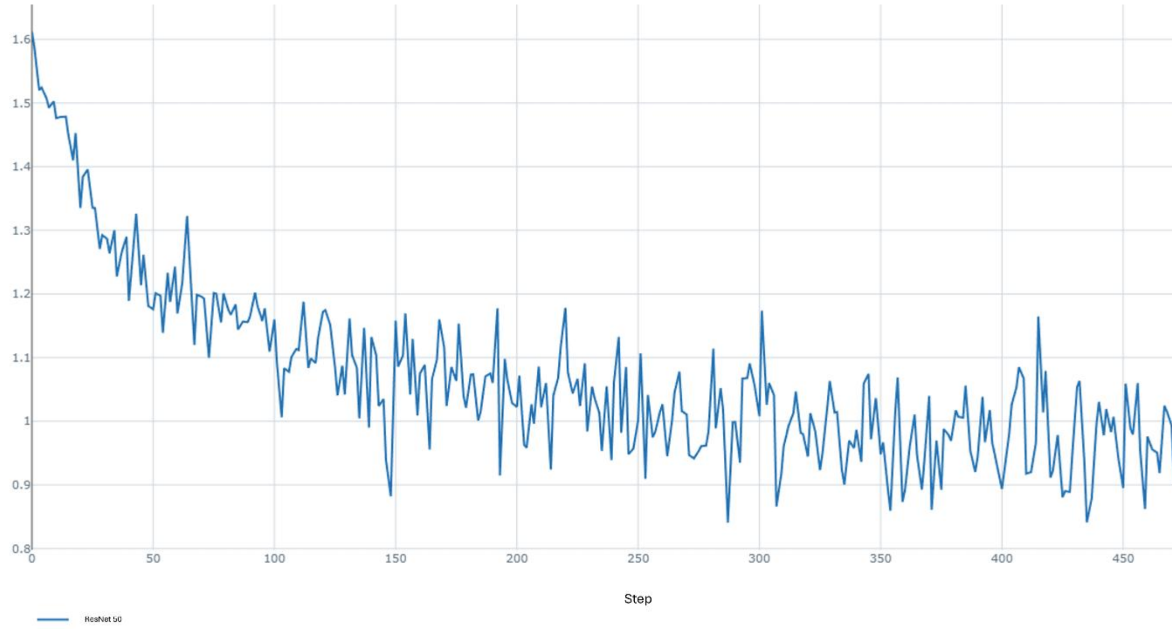

1.6
1.5
1.4
1.3
1.2
1.1
1
0.9
0.8
0
50
100
150
200
250
300
350
400
450
Step
ResNet 50

(a)

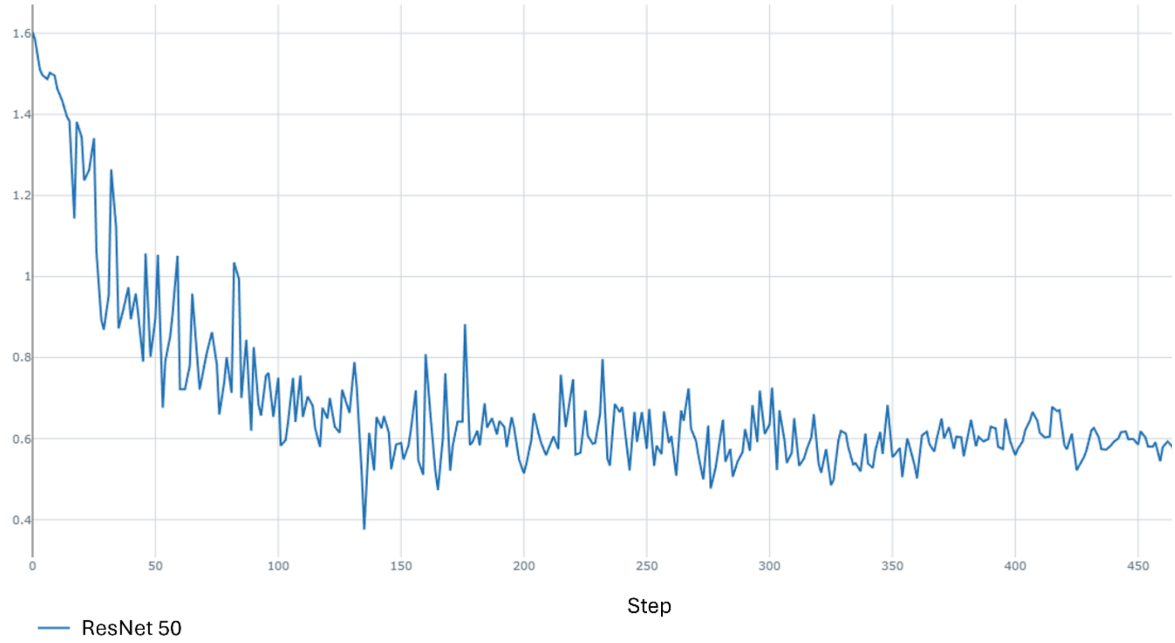

1.6
1.4
1.2
1
0.8
0.6
0.4
0
50
100
150
200
250
300
350
400
450
Step
ResNet 50

(b)

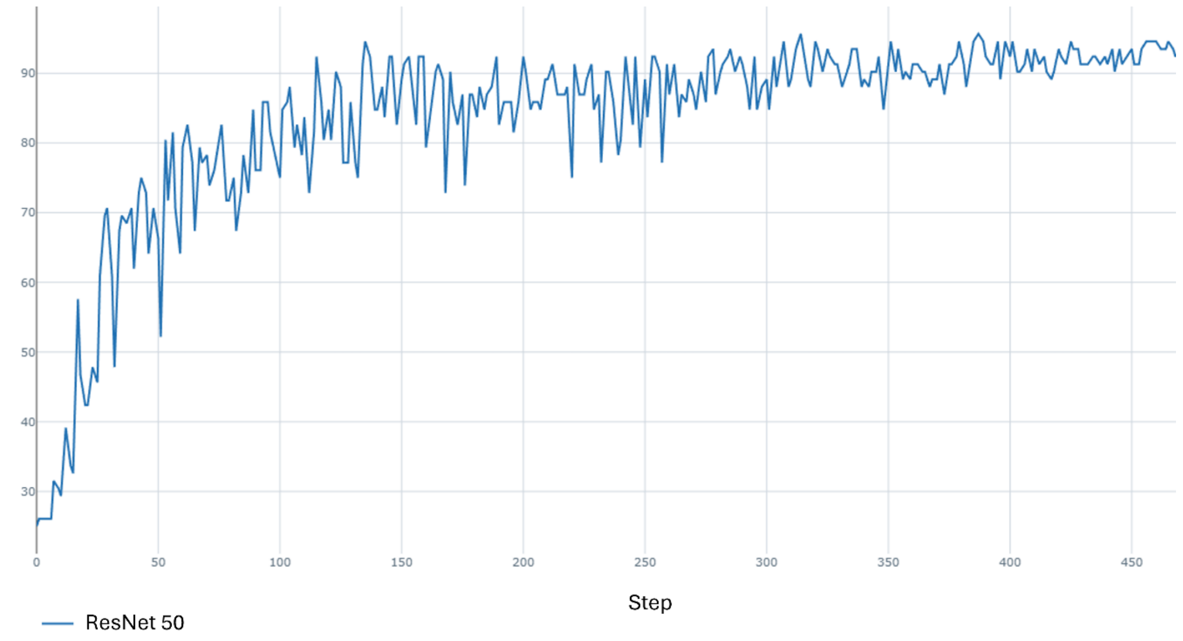

(c)

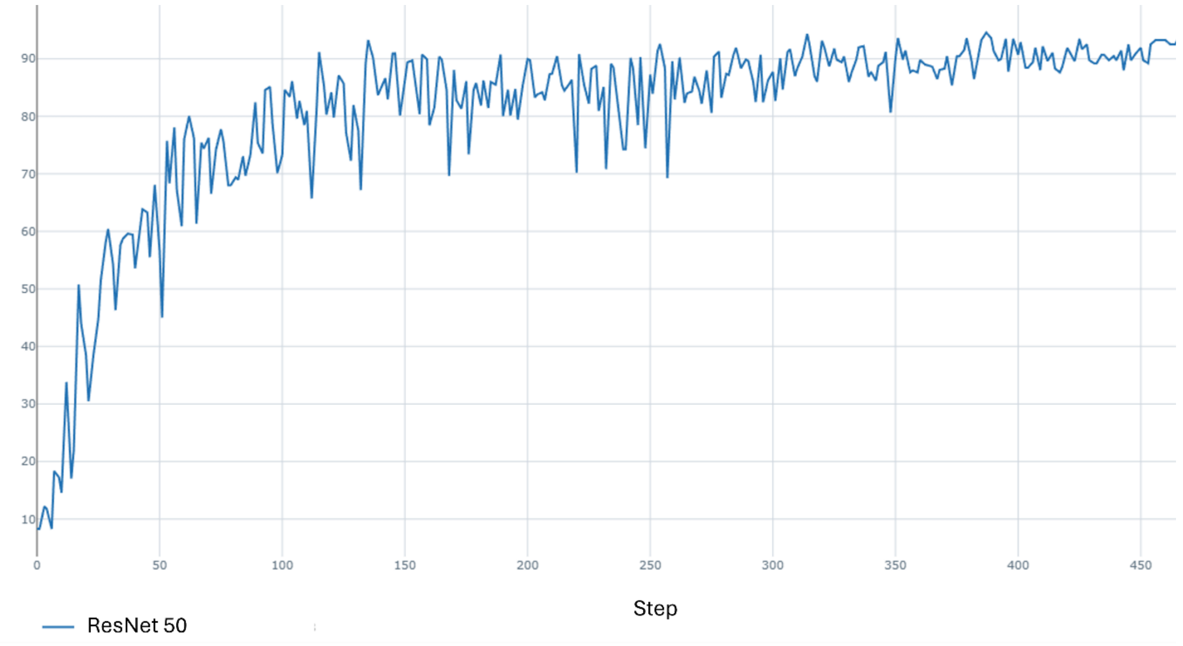

(d)

Fig. S3 Learning curves across training for the vendor classification model on the augmented dataset using five fold cross validation: (a) Training loss over epochs; (b) Validation loss over epochs; (c) Validation accuracy over epochs; (d) Validation F1 score over epochs.

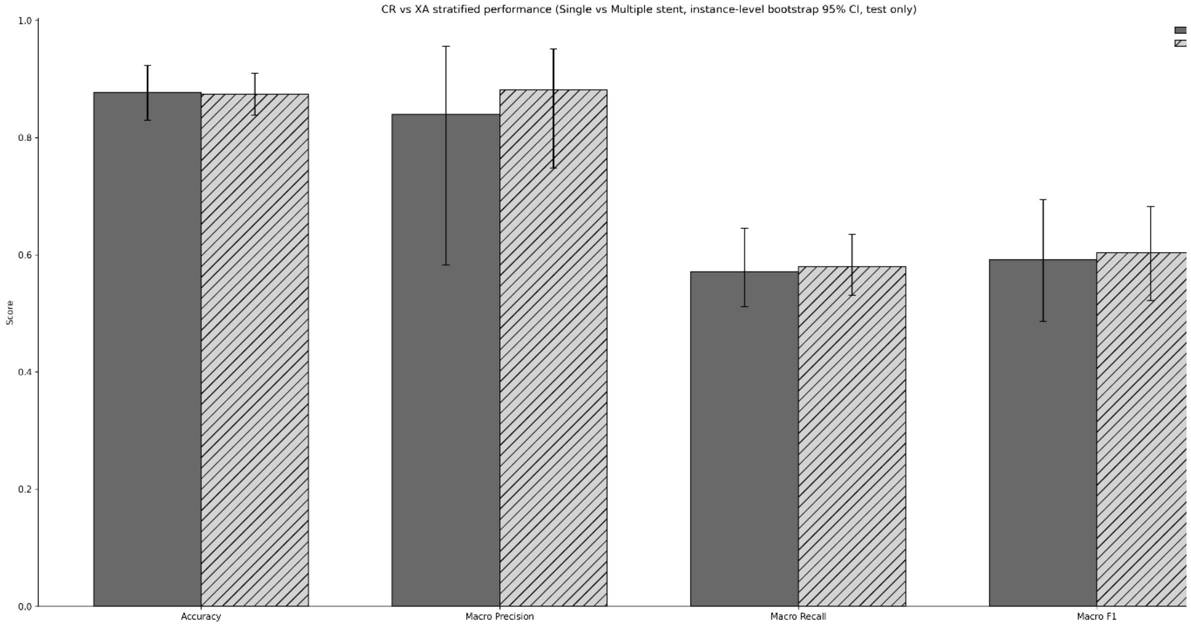

CR vs XA stratified performance (Single vs Multiple stent, instance-level bootstrap 95% CI, test only)
Score
1.0
0.8
0.6
0.4
0.2
0.0
Accuracy
Macro Precision
Macro Recall
Macro F1

(a)

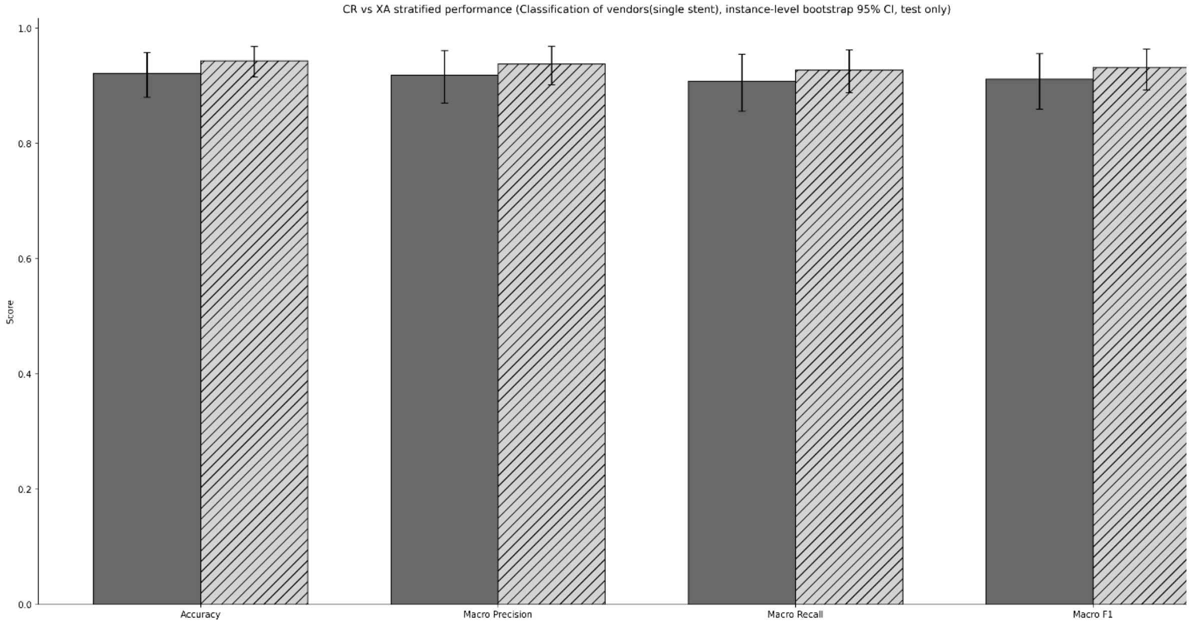

CR vs XA stratified performance (Classification of vendors(single stent), instance-level bootstrap 95% CI, test only)
Score
1.0
0.8
0.6
0.4
0.2
0.0
Accuracy
Macro Precision
Macro Recall
Macro F1

(b)

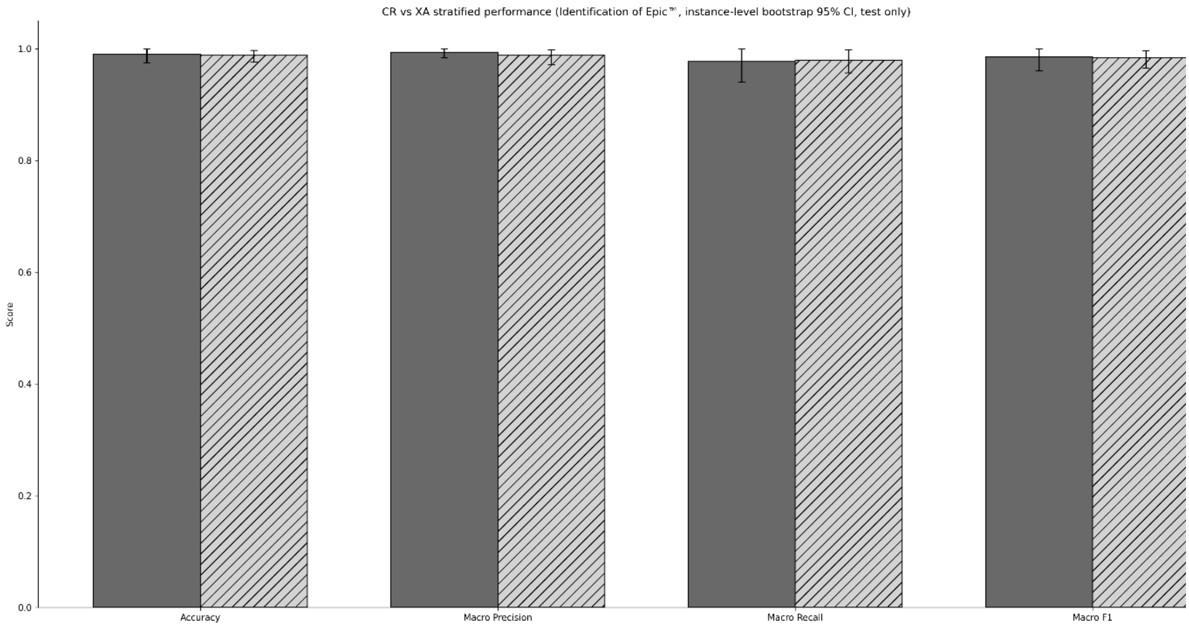
CR vs XA stratified performance (Identification of Epic™, instance-level bootstrap 95% CI, test only)
Score
0.0
0.2
0.4
0.6
0.8
1.0
Accuracy
Macro Precision
Macro Recall
Macro F1

(c)

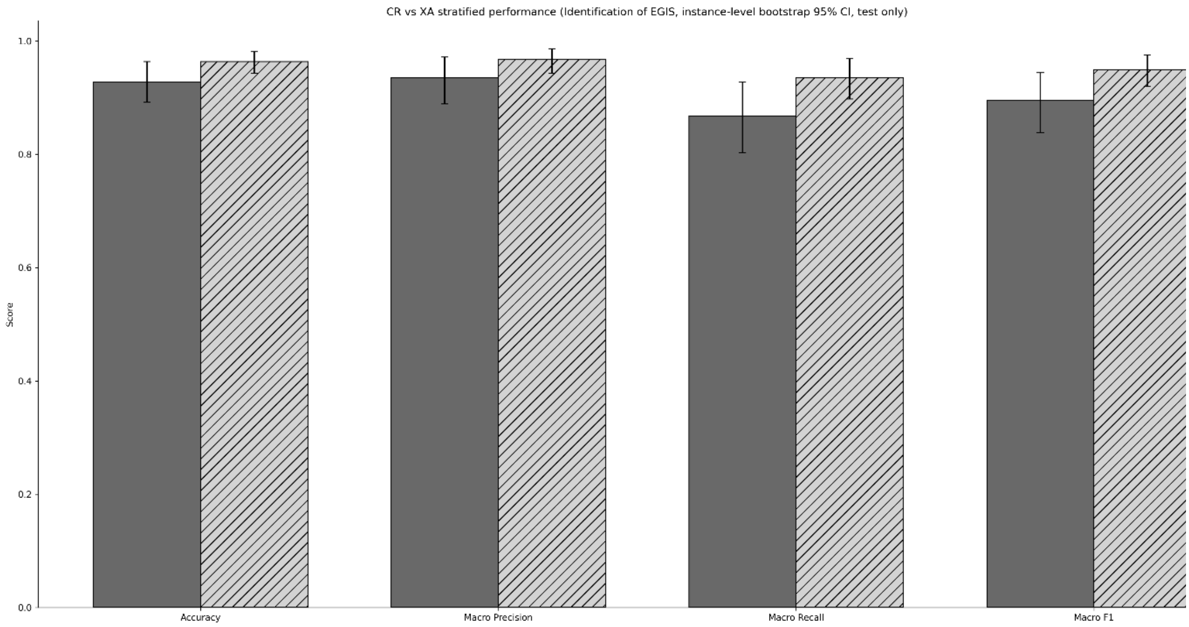
CR vs XA stratified performance (Identification of EGIS, instance-level bootstrap 95% CI, test only)
Score
0.0
0.2
0.4
0.6
0.8
1.0
Accuracy
Macro Precision
Macro Recall
Macro F1

(d)

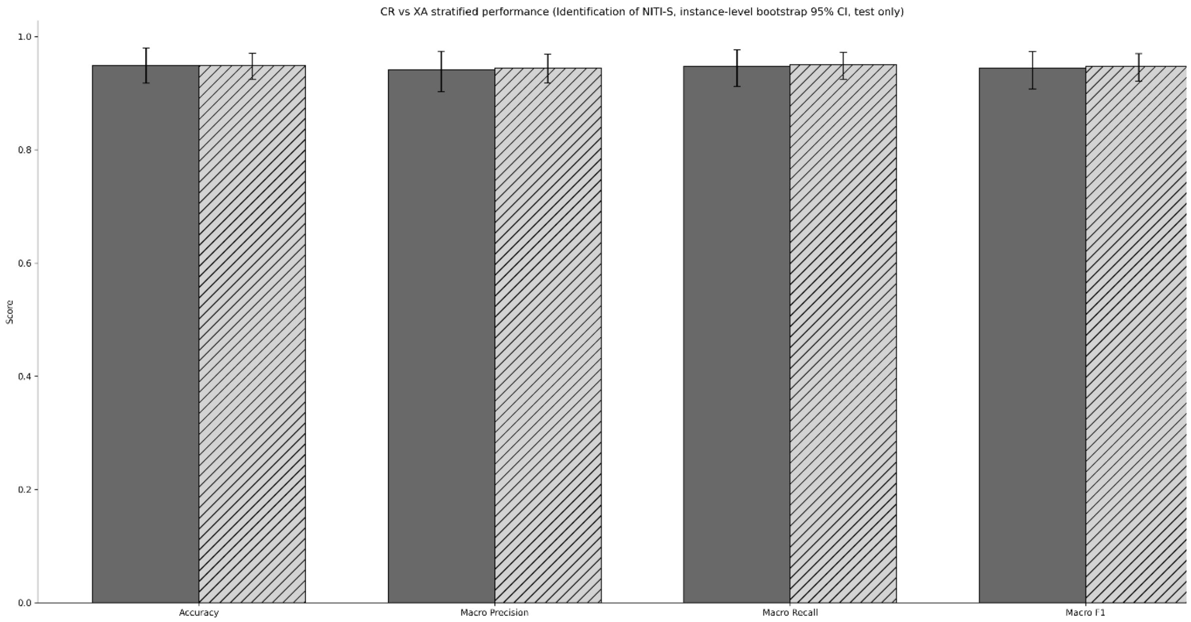
CR vs XA stratified performance (Identification of NITI-S, instance-level bootstrap 95% CI, test only)
Score
1.0
0.8
0.6
0.4
0.2
0.0
Accuracy
Macro Precision
Macro Recall
Macro F1

(e)

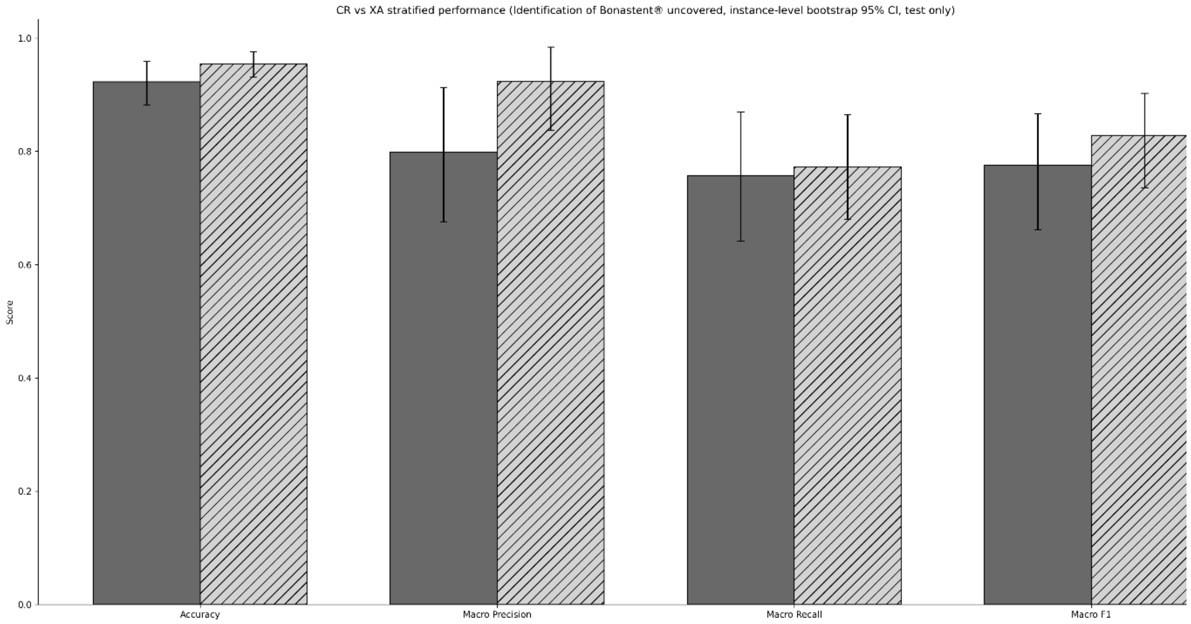
CR vs XA stratified performance (Identification of Bonastent® uncovered, instance-level bootstrap 95% CI, test only)
Score
1.0
0.8
0.6
0.4
0.2
0.0
Accuracy
Macro Precision
Macro Recall
Macro F1

(f)

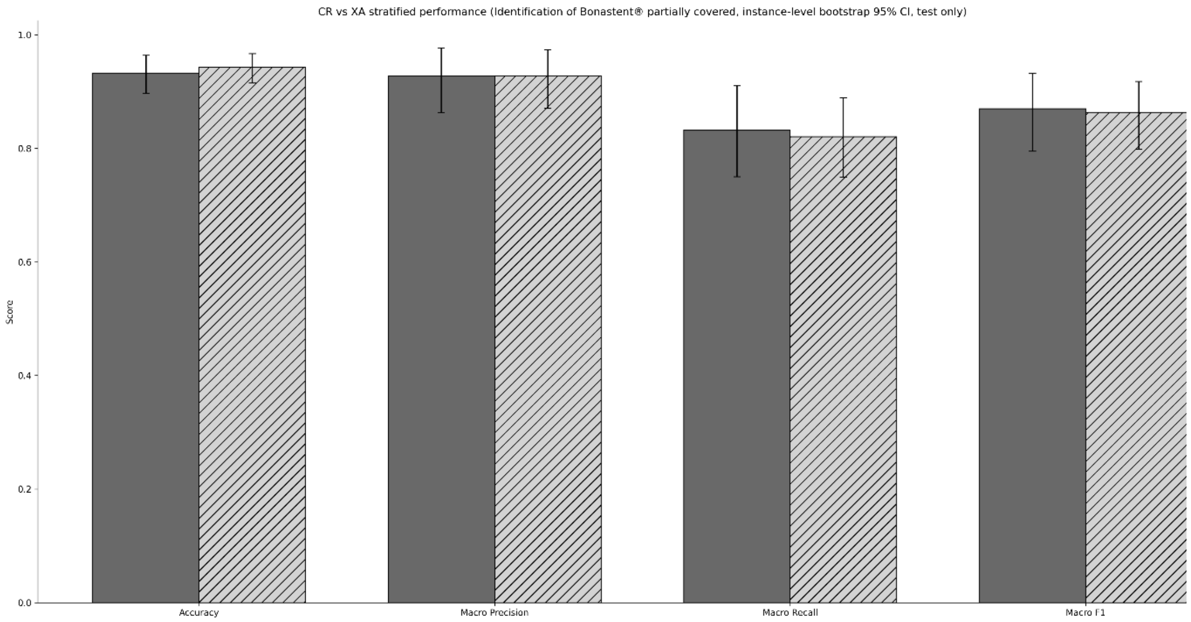

(g)

Fig. S4 Stratified analysis of model performance by acquisition modality (fluoroscopic x-ray *versus* computed radiography): (a) Single versus multiple stents; (b) Vendor classification; (c) Epic™ identification; (d) EGIS identification. (e) Niti S identification; (f) Bonastent® uncovered identification; (g) Bonastent® partially covered identification.

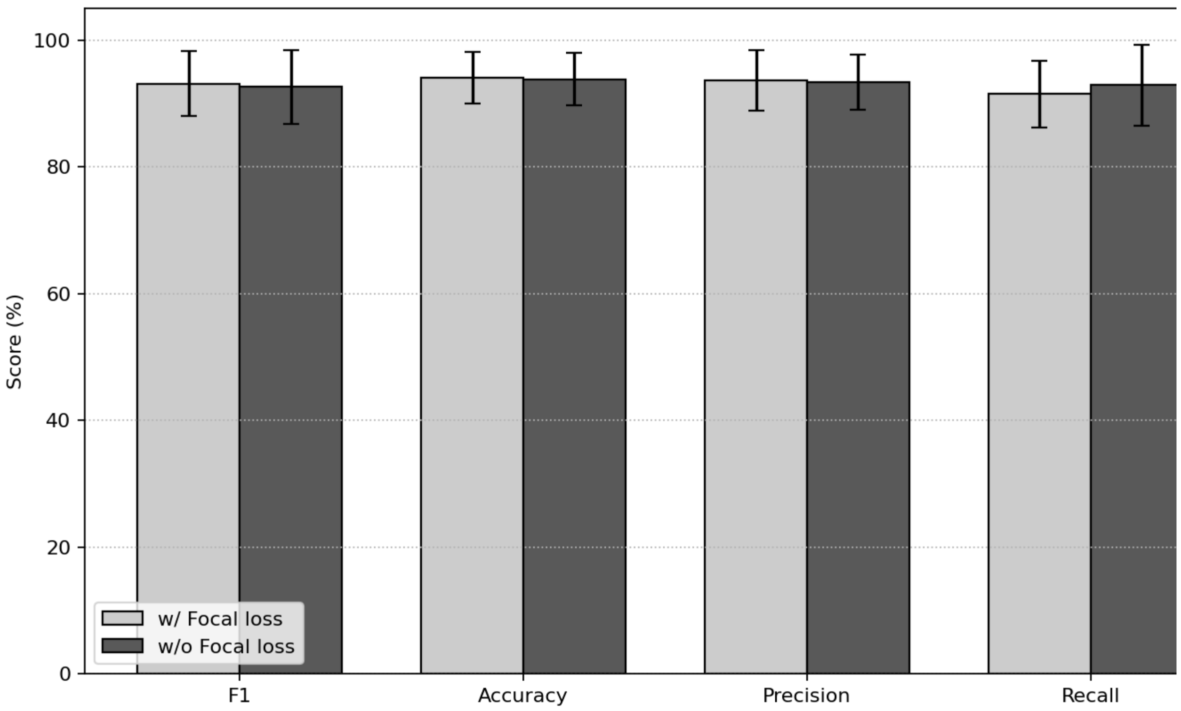

Fig. S5 Focal loss ablation for vendor classification on the augmented dataset. Comparison of vendor classification performance trained with and without focal loss under identical hyperparameters for 500 epochs using five fold cross validation.

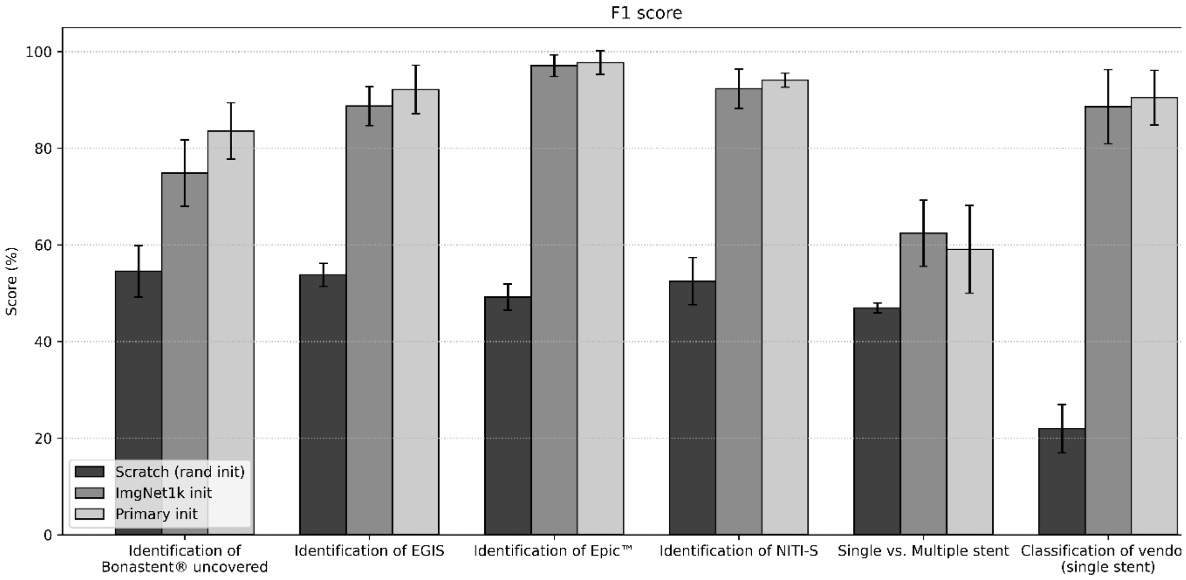

(a)

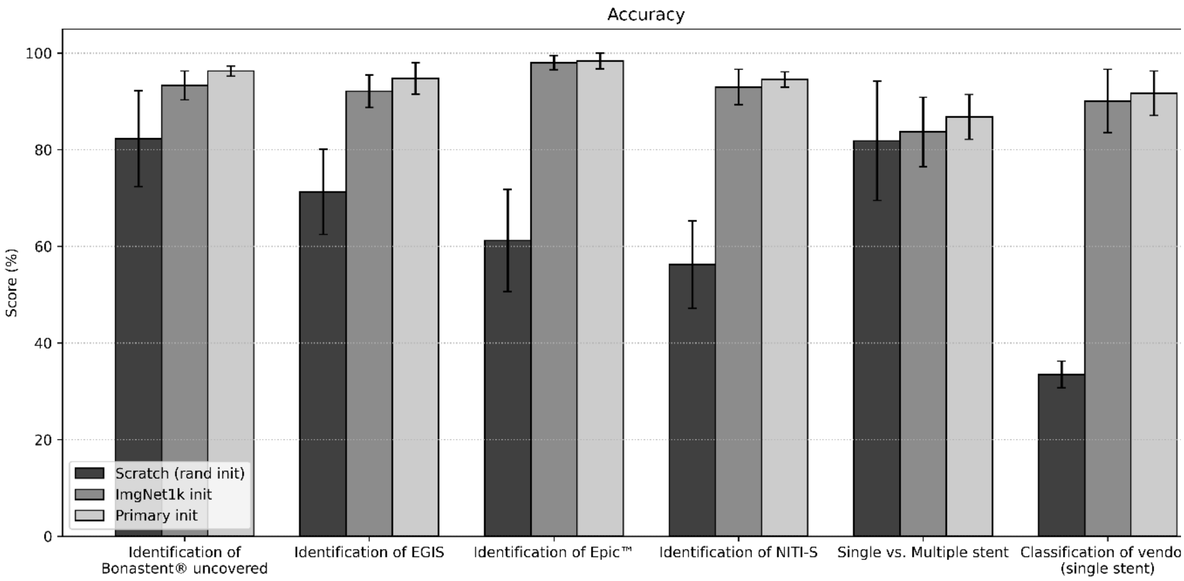
Accuracy
Score (%)
0
20
40
60
80
100
Scratch (rand init)
ImgNet1k init
Primary init
Identification of Bonastent® uncovered
Identification of EGIS
Identification of Epic™
Identification of NITI-S
Single vs. Multiple stent
Classification of vendo
(single stent)

(b)

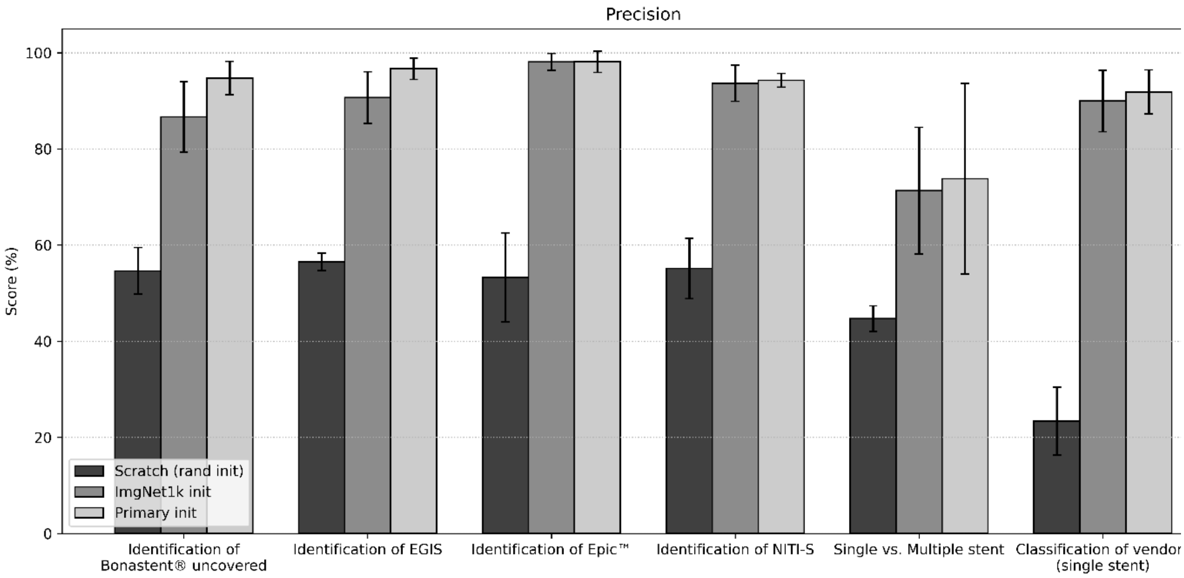
Precision
Score (%)
0
20
40
60
80
100
Scratch (rand init)
ImgNet1k init
Primary init
Identification of Bonastent® uncovered
Identification of EGIS
Identification of Epic™
Identification of NITI-S
Single vs. Multiple stent
Classification of vendor
(single stent)

(c)

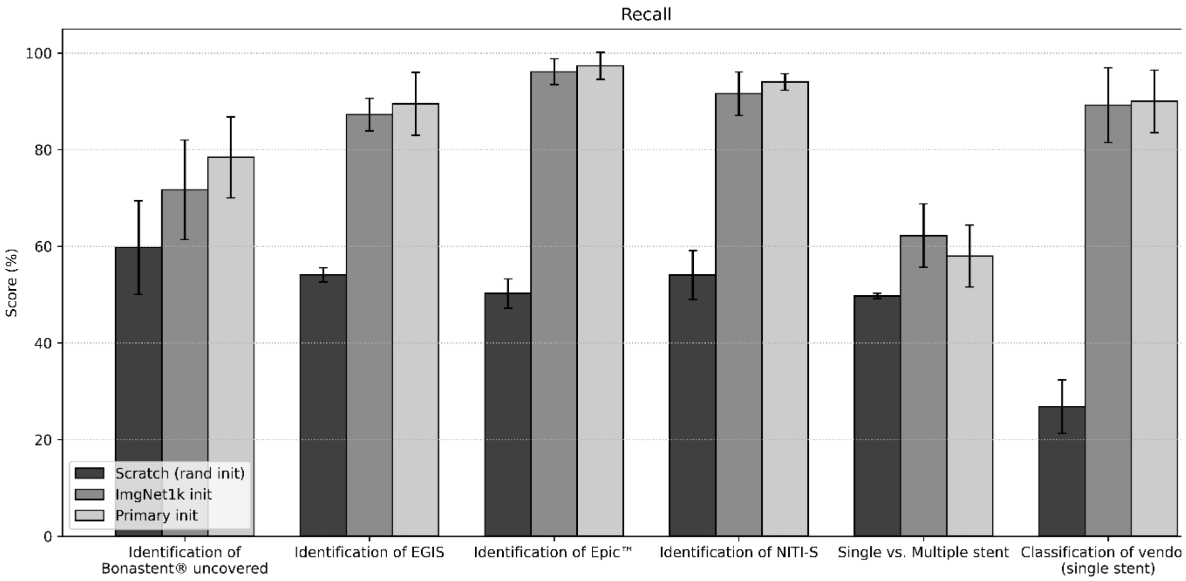

(d)

Fig. S6 Comparison of initialization strategies across tasks**:** (a) Macro F1 score. (b) Accuracy. (c) Macro precision. (d) Macro recall. Scores are reported as mean ± standard deviation across five sets for each task under three initialization strategies: From Scratch (random initialization), Transfer learning (ImgNet init), and Transfer learning (Primary init).
